# Immune-Related Transcriptome of *Coptotermes formosanus* Shiraki Workers: The Defense Mechanism

**DOI:** 10.1371/journal.pone.0069543

**Published:** 2013-07-16

**Authors:** Abid Hussain, Yi-Feng Li, Yu Cheng, Yang Liu, Chuan-Cheng Chen, Shuo-Yang Wen

**Affiliations:** Department of Entomology, College of Natural Resources and Environment, South China Agricultural University, Guangzhou, P. R. China; Onderstepoort Veterinary Institute, South Africa

## Abstract

Formosan subterranean termites, *Coptotermes formosanus* Shiraki, live socially in microbial-rich habitats. To understand the molecular mechanism by which termites combat pathogenic microbes, a full-length normalized cDNA library and four Suppression Subtractive Hybridization (SSH) libraries were constructed from termite workers infected with entomopathogenic fungi (*Metarhizium anisopliae* and *Beauveria bassiana*), Gram-positive *Bacillus thuringiensis* and Gram-negative Escherichia coli, and the libraries were analyzed. From the high quality normalized cDNA library, 439 immune-related sequences were identified. These sequences were categorized as pattern recognition receptors (47 sequences), signal modulators (52 sequences), signal transducers (137 sequences), effectors (39 sequences) and others (164 sequences). From the SSH libraries, 27, 17, 22 and 15 immune-related genes were identified from each SSH library treated with *M. anisopliae*, *B. bassiana*, *B. thuringiensis* and *E. coli*, respectively. When the normalized cDNA library was compared with the SSH libraries, 37 immune-related clusters were found in common; 56 clusters were identified in the SSH libraries, and 259 were identified in the normalized cDNA library. The immune-related gene expression pattern was further investigated using quantitative real time PCR (qPCR). Important immune-related genes were characterized, and their potential functions were discussed based on the integrated analysis of the results. We suggest that normalized cDNA and SSH libraries enable us to discover functional genes transcriptome. The results remarkably expand our knowledge about immune-inducible genes in *C. formosanus* Shiraki and enable the future development of novel control strategies for the management of Formosan subterranean termites.

## Introduction

The Formosan subterranean termite, *Coptotermes formosanus* Shiraki (Isoptera: Rhinotermitidae) is a serious pest that infests economically important crops and structures such as houses, buildings, boats, utility poles and underground telephone cables [1]. This termite species has been estimated to cause damage valued at one billion dollars annually in the United States of America [2] and 0.8 billion RMB in the People’s Republic of China [3]. Entomopathogenic fungi such as *Metarhizium anisopliae* and *Beauveria bassiana* have received particular attention as the basis of an alternate termite management strategy because these termites live in soil that is also favorable for fungal growth [4]. The last 40 years intensive research clearly states that biological control agents are effective only in the lab [1]. The failure of entomopathogenic fungi in the field to control termites led researchers to study their defense mechanisms. Recently, Hussain et al. [1] reviewed the reasons for the limited success of entomopathogenic fungi in controlling colonies of subterranean termites and proposed that *C. formosanus* Shiraki colonies have evolved main three defense mechanisms (behavioral, biochemical and physiological) that reduce the incidence of fungal epizootics within their colonies. Five behavioral and biochemical adaptation mechanisms are involved in resistance to fungal infection: pathogen alarm behavior based on volatile organic compounds (VOCs) that warns foragers about the presence of lethal fungi and causes them to avoid infected areas; biochemical protection by the nestmates, who continuously secrete high amounts of toxic compounds, including naphthalene, *n*-hexanoic acid and nonanal, into the closed environment of the nest, walling-off infected areas of the colony, mutual grooming among nestmates, and removal of fungal-infected termites [1], [5]–[8].

Insects have an efficient and potent innate immune system including humoral and cellular immune responses to protect themselves from microbial infection [9]. In recent years, significant progress has been made in studying the immune mechanism at the molecular level in insects, for instance, *Apis mellifera* [10], *Bombyx mori* [11], *Drosophila melanogaster* [9], and *Tribolium castaneum* [12]. However, the immune related genes and their families in termites are not well studied. In the past, small scale investigations tried to explore the immune related gene diversity. During 2003, the suppression subtractive hybridization procedure was used to explore genes expressed in *Mastotermes darwiniensis* following exposure to *M. anisopliae* (strain FI-1248, CSIRO Entomology Canberra). The results revealed the up-regulation of *transferring* gene [13]. Bulmer and Crozier [14] investigated the molecular evolution of three immune related genes including Gram-negative binding protein 1 (GNBP1), GNBP2 and Relish in 13 Australian termite species (*Nasutitermes*). Their comparative results indicated that Relish, a transcription factor has experienced greater selective pressure to change the composition of amino acid relative to GNBPs. Later on, significant reduction in fungal germination was observed from the eluted proteins extracted from *Zootermopsis angusticollis* infected with *M. anisopliae* that might involve in immune response [15]. In another study, primary reproductives were exposed to pathogens to investigate their impacts on the immunity and reproduction. Their findings revealed that the reproduction can reduce the immune response among female primary reproductives [16]. The cellular immune system of *Z. angusticollis* was explored by studying the types of hemocytes among dampwood termites exposed with *M. anisopliae*. They identified three types of hemocytes. They suggested that the reduction in hemocytes number directly related with the appearance of hyphal bodies. Moreover, the invading conidia of *M. anisopliae* overtake the cellular immune response by destroying hemocytes of *Z. angusticollis* [17]. More recently, 182 expressed sequence tags (ESTs) were obtained by suppression subtractive hybridization from *Reticulitermes flavipes* infected with *M. anisopliae*. Because of high rebundancy all the ESTs were assembled into 19 clusters. Their library revealed the identification of only 9 immune related genes [18].

Most of these insects, including termites, actively defend themselves against fungal infections and are resistant to microbial infections. Their defense system mainly consists of various innate immune reactions, including phagocytosis [19], the activation of proteolytic cascades leading to localized melanization and coagulation [20], and the synthesis of potent antimicrobial peptides (AMPs) by the fat body [21]. These unique type AMPs released into the hemolymph, where they synergistically act to destroy harmful invaders such as bacteria, fungi, and protozoa [22].

Most knowledge of genes involved in the immune response has been obtained from studies of other insects, and knowledge about the immune response of Formosan subterranean termites is in its infancy. Recently, we have attempted to investigate the role of immune reactions in disease resistance. Our results suggested that exposure to entomopathogenic fungi (*Metarhizium anisopliae* strain 2049 and *Beauveria bassiana* strain 3005) and bacteria (*Bacillus thuringiensis* and *Escherichia coli*) greatly induce immune reactions that significantly increase the resistance in their homogenates to subsequent challenge. Furthermore, our findings illustrate that the pattern of the antimicrobial responses of the homogenates varied with the post-inoculation time. We might assume that the nature of the antimicrobial response at different time intervals might be due to the presence of several antimicrobial peptides [23].

However, the molecular mechanisms that regulate immune responses in *C. formosanus* Shiraki are largely unknown. In the present study, a normalized cDNA library and four SSH libraries obtained from whole body homogenates of immunized *C. formosanus* Shiraki workers, which were infected with entomopathogenic fungi (*M. anisopliae* and *B. bassiana*), Gram-positive *B. thuringiensis* and Gram-negative E. coli, were constructed and analyzed to determine potential immune-related genes that respond to different microbes. The expressive pattern of important immune-related genes was further investigated using q-PCR to confirm the involvement of these genes in termite immunity in various microbial infections.

## Materials and Methods

### 2.1 Ethics Statement

N/A.

### 2.2 Collection and Maintenance of Termites

Workers and the soldiers of *C. formosanus* Shiraki were collected from Huolu Shan Forest Park, approximately 3.5 kilometers from the campus of South China Agricultural University, Guangzhou, P. R. China. No specific permits were required for collecting termites from this public park. Termites were maintained at temperatures between 24°C and 27°C in plastic buckets containing pine wood stakes (*Pinus* sp.) placed over moist sterile soil in complete darkness [24]. Termites were reared by keeping the ratios of the workers and the soldiers same as they were present in the field.

### 2.3 Fungal and Bacterial Biocontrol Agents

Both fungal strains (*B. bassiana* strain EBCL 03005 and *M. anisopliae* strain EBCL 02049) were produced in Petri dishes on potato dextrose agar (PDA) (Difco Laboratories, Detroit, MI, USA). The fungi were incubated for 24 days at 25±0.5**°**C in complete darkness. The conidia were harvested in 0.05% Tween 80 solution (Sigma). The concentration (1 ×10^6^ conidia/ml) and viability (>95%) of the fungal strains were calculated as described in detail by Hussain et al. [5]. The Gram-positive bacterial strain *B. thuringiensis* var. *galleriae* and the Gram-negative bacterial strain E. coli K12D31 were maintained on Luria Bertani (LB) medium. The bacterial cultures were maintained in an incubator at 37±0.5**°**C in complete darkness before use in the experiment. The bacterial suspensions were adjusted to 1 ×10^6^ colony forming units (cfu) ml^−1^ by using sterile distilled water.

### 2.4 Insect Immunization

The termite workers from four different colonies were immunized by immersing them in each fungal and bacterial strain suspension separately in micro-centrifuge tubes with gentle swirling for 5 s as described in our previous paper [23]. We have used swirling method instead of typical immune induction methods (injection or feeding on infected food). In our preliminary study, we tried to induce the immune response among workers by injection. However, injection could not induce immune response (growth inhibition) instead caused 100% mortality within 36 h. The second possible option was to infect the workers with food impregnated with microbes. We could not use this method because the termites had the ability to detect the microbes and tried to increase their distance from the pathogens [5]. Recently, Wright and Cornelius [25] also used the same method to infect the *C. formosanus* with microbes especially *B. thuringiensis* strain 33679. By keeping in mind the failure of microbes to induce termite immune response by injection and feeding, swirling method that produced encouraging results was used to induce immune response among *C. formosanus* Shiraki workers. The exposed workers were allowed to dry on Whatman no. 1 filter paper. Control workers were kept uninfected, immersed them into 0.04% Tween 80 (Sigma) and water in case of control treatment for fungi and bacteria, respectively. The immunized and uninfected (Control) workers were maintained in groups in Petri dishes (95 × 15 mm), containing dampened filter paper. The termites were incubated at 26±0.5°C and 85±5% RH until sampling. Based on our previous results, we used only one colony for the construction cDNA libraries because we could not find significant differences in immune response from workers belonging to different colonies. The termites were collected at specific times after infection with *M. anisopliae* (12, 18, 24, 42, 48, 54, 66, 72 and 78 h), *B. bassiana* (6, 12, 18, 30, 36, 42, 48, 60 and 66 h), *B. thuringiensis* (12, 18, 24, 42, 48 and 54 h) and *E. coli* (6, 54, 60 and 66 h), and frozen immediately in liquid nitrogen and stored at −86°C. The specific times used for each agent were chosen based on our previous investigations [23].

### 2.5 Construction of the Normalized cDNA Library

Seven workers from each group that was treated at the specific time intervals listed above were frozen using liquid nitrogen and finely pulverized. Total RNA was extracted using Trizol reagent (Invitrogen, USA) according to the manufacturer’s specifications from the whole bodies of *C. formosanus* Shiraki workers. The guts were not removed because in the preliminary antimicrobial activity assays (growth inhibition) we also observed bacterial growth inhibition from the gut homogenates. mRNA was obtained from total RNA using the Oligotex mRNA Mini Kit (Qiagen, Hilden, Germany) according to the manufacturer’s protocol. The integrity of the total RNA and mRNA was verified using agarose gels, and their quantity and purity were determined spectrophotometrically. For the first-strand cDNA synthesis, purified mRNA was denatured in the presence of 3′ SMART CDS Primer II A (12 µM) at 72°C for 3 min in an RNAse-free tube, rapidly chilled on ice, mixed with 5× first-strand buffer, 100 mM DTT, 10 mM dNTP Mix, SMARTer II A Oligonucleotide (12 µM), RNAse Inhibitor, and SMARTScribe™ Reverse Transcriptase (100 U). First strand cDNA was synthesized at 42°C for 90 min, and the tubes were placed on ice according to the instructions of the In-Fusion™ SMARTer™ cDNA library construction kit (Clontech Laboratories, Inc.). For the second-strand cDNA synthesis, deionized H_2_0, 10× Advantage 2 PCR buffer, 50× dNTP Mix (10 mM), 5′ PCR Primer II A (12 µM), and 50X Advantage 2 Polymerase mix were mixed with the first strand. The mixture was then amplified using long distance (LD) PCR. The optimal numbers of cycles for each sample were determined according to the manufacturer’s protocol. The amplified double strand (ds) cDNAs, after purification with DNA fragment purification kit (Clontech Laboratories, Inc.), were used to construct the normalized cDNA library.

The normalized cDNA library was constructed using an In-Fusion™ SMARTer™ cDNA library construction kit (Clontech Laboratories, Inc.) combined with a Trimmer-direct cDNA Normalization kit (Evrogen). The purified ds cDNA was first denatured at 98°C for 2 min and allowed to renature at 68°C for 5 h, then 1/4 Duplex-specific nuclease (DSN) was used to further reassociate ds cDNA for 25 min at 68°C. This normalized ds cDNA was amplified using an Evrogen PCR M1 5′-AAGCAGTGGTATCAACGCAGAGT- 3′ primer under the following conditions: 95°C for 1 min, then 10 cycles of 95°C for 15 s, 66°C for 20 s, and 72°C for 3 min. A second amplification was then performed using an Evrogen PCR M2 (5′-AAGCAGTGGTATCAACGCAG-3′) primer under the following conditions: 95°C for 1 min, followed by 12 cycles of 95°C for 15 s, 64°C for 20 s, and 72°C for 3 min. The products were finally extended at 64°C for 15 s and 72°C for 3 min. The amplified PCR products were purified according to the instructions included with the CHROMA SPIN™ DEPC-1000 Column kit (Clontech Laboratories, Inc.). Finally, the ds cDNA was ligated into the pSMART2IF Linearized vector supplied in the kit. The ligation products were electroporated into electrocompetent *E. coli* (strain DH5a) cells (TaKaRa Biotech) using a Bio-Rad Gene Pulser II Electroporator at 1.6 kV. The transformed cells were recovered in LB medium by shaking at 220 rpm and 37°C for 1 h. The titer of the primary library was calculated according to the protocol included with the In-Fusion™ SMARTer™ cDNA library construction kit. The original library was amplified by spreading on 100 plates of LB agar supplemented with 100 µl/ml ampicillin (Sigma), 1 mM IPTG (isopropyl-b-D-thiogalactopyranoside) (Sigma) and 75 µg/ml X-Gal (5-bromo-4-chloro-3-indolyl-b-D-galactopyranoside) (Sigma), and culturing overnight at 37°C. Colonies were collected and stored in 25% glycerol at −80°C.

To identify the cDNA inserts, clones were randomly selected for PCR amplification. PCR was performed with a vector-specific primer under the following conditions: 95°C for 1 min, followed by 35 cycles of 95°C for 15 sec, 64°C for 20 sec and 72°C for 3 min, with a final extension at 72°C for 3 min. The products were analyzed using 1.2% agarose gel electrophoresis.

Three thousand white colonies from the normalized cDNA library of immunized *C. formosanus* workers were sequenced using the M13 primer provided by the Invitrogen Biotechnology Company, Guangzhou, China. Unique DNA sequences were compared against non-redundant nucleotide and protein databases using BLASTx with an expectation (E) value cutoff of 10^−5^ using the powerful and free data mining tool Blast2GO [26].

### 2.6 Construction of Suppression Subtractive Hybridization Libraries

Total RNA samples for each subtracted library from infected (*M. anisopliae, B. bassiana, B. thuringiensis* and *E. coli*) and uninfected (Control) *C. formosanus* workers at each time interval were separately extracted using Trizol reagent (Invitrogen). Total RNA derived from each treatment (*M. anisopliae, B. bassiana, B. thuringiensis* and *E. coli*) at different time intervals were pooled. Poly (A)^+^ was purified using the Oligotex mRNA Mini Kit (Qiagen, Hilden, Germany) according to the manufacturer’s protocol. The integrity of the total RNA and messenger RNA was checked on an agarose gel, and their quantity and purity were determined spectrophotometrically.

The mRNA for each library was then separately reverse-transcribed and amplified to produce high-quality complementary DNA (cDNA) from a small sample using the SMARTer™ PCR cDNA Synthesis Kit (Clontech Laboratories, Inc.) according to the user manual. Briefly, the mRNA was denatured by mixing with SMART CDS primer IIA (12 µM) at 72°C for 3 min; then, the temperature was reduced to 42°C for 2 min and the first strand buffer, DTT, dNTP, SMARTer II A Oligonucleotide, RNase Inhibitor and SMARTScribe™ Reverse Transcriptase were added as instructed by the manufacturer. The denatured mRNA was then reverse transcribed for 10 min at 70°C to synthesize single-stranded (ss) cDNA. The resulting ss cDNA was used as a template for the PCR amplification of ds cDNA using the Advantage cDNA PCR Kit (Clontech Laboratories, Inc.). For the ds cDNA synthesis, ss cDNA was combined with 10× Advantage 2 PCR Buffer, 50× dNTP, 5′ PCR Primer II A, 50× Advantage 2 polymerase mix and deionized water. The mixture was then amplified using LD PCR. The optimal number of cycles for each sample was determined according to the manufacturer’s protocol. The PCR products were used to construct the SSH cDNA libraries.

The ds cDNAs for each library were digested after purification using the restriction enzyme Rsa I to create shorter blunt-ended fragments, which are necessary for adaptor ligation according to the manufacturer’s instructions included in the PCR-Select™ cDNA Subtraction Kit (Clontech Laboratories, Inc.). The Rsa I-digested cDNA fragments were purified using phenol–chloroform–isoamyl alcohol (25∶24∶1). The purified RsaI-digested cDNAs were precipitated using 4 M NH_4_OAc and 95% ethanol. The cDNA pellet was washed in 80% ethanol, dissolved in H_2_O and stored at −20°C until further use. For the SSH procedure, the digested cDNA from *C. formosanus* workers infected with the microbes was designated as the tester (experimental), and the digested cDNA from uninfected *C. formosanus* workers was designated as the driver (reference).

The purified digested tester cDNA was diluted and ligated with Adaptor 1∶5′-CTAATACGACTCACTATAGGGCTCGAGCGGCCGCCCGGGCAGGT-3′ and Adaptor 2R: 5′-CTAATACGACTCACTATAGGGCAGCGTGGTCGCGGCCGAGGT-3′) at the 5′ - end of each strand in separate ligation reactions at 16°C overnight using T4 DNA ligase. Subsequently, the adaptor 1-ligated and adaptor 2R-ligated tester cDNAs for each library were separately hybridized at 68°C for 8 h with an excess of Rsa-I digested driver cDNA after denaturation at 98°C for 90 s in a thermal cycler. After the first hybridization reaction, the two samples of each library were mixed together, fresh Rsa I digested driver cDNA was added, and the mixture was hybridized again at 68°C overnight to further enrich the differentially expressed sequences. The resulting mixture was diluted and amplified by two rounds of suppression PCR to enrich desired cDNA (i.e., containing both adaptors) through exponential amplification using the Advantage® cDNA PCR Polymerase Mix Kit (Clontech Laboratories, Inc). The primary PCR with primer 1 was carried out as follows: denaturation at 94°C for 25 s, followed by 27 cycles of 94°C for 10 s; 66°C for 30 s and 72°C for 1.5 min. Secondary PCR using the nested primers 1 and 2R was performed on the diluted primary PCR products for 12 cycles using the following parameters: 94°C for 30 s; 68°C for 30 s, and 72°C for 90 s. The amplified PCR products were purified according to the instructions included with the DNA Fragment Purification Kit (Clontech Laboratories, Inc). Finally, the resulting purified cDNAs were cloned into the pMD®20-T vector (TaKaRa Biotech.) and transformed into *E*. *coli* (DH5α; Invitrogen) competent cells to construct four SSH libraries by plating onto Luria–Bertani (LB) agar plates supplemented with 100 µl/ml ampicillin, 1 mM IPTG (isopropyl-b-D-thiogalactopyranoside) and 75 µg/ml X-Gal (5-bromo-4-chloro-3-indolyl-b-D-galactopyranoside); the plates were incubated overnight at 37°C. Insert size was checked using the PCR amplification kit (Invitrogen) and vector specific M13 primers; the conditions used were as follows: 94°C for 5 min, followed by 30 cycles of 94°C for 30 sec, 50°C for 45 sec and 72°C for 1 min, followed by extension at 72°C for 10 min. β-actin was used as reference gene. Samples of the PCR products (5 µl) were analyzed using 1.2% agarose gel electrophoresis.

### 2.7 Expression Analysis of Specific Immunity-related Genes by Quantitative Real-time PCR (qPCR)

Total RNAs were extracted separately from the whole body homogenates of *C. formosanus* workers immunized with the suspensions of *M. anisopliae*, *B. bassiana*, *B. thuringiensis* and *E. coli*. The total RNA obtained for each treatment group was reverse transcribed using the ReverTra Ace® qPCR RT Kit (FSQ-101; Toyobo). The RNA was quantified using the CFX96 Real-Time System (Bio-Rad). All primer sets were designed from our EST database using gene script (https://www.genscript.com) as listed in Table 1. The reaction mixture (20 µl) included 1 µl cDNA, 0.4 µl of each primer (10 µM), 10 µl of SYBR Premix Ex Tag (TaKaRa Biotech.) and 8.2 µl ddH_2_O. The PCR cycling parameters used were 95°C for 30 s, followed by 40 cycles of 95°C for 5 s and 60°C for 30 s. All individual PCR reactions were repeated three times. The comparative quantitation method (ΔΔCt) was used to compare different treatments, and the obtained results were transformed to absolute values with 2^−ΔΔCt^ to obtain relative fold expressions compared to the control treatment [27]. Relative fold expressions for each gene were set to 1 for the control treatment (the calibrator). Data were analyzed using analysis of variance (ANOVA), and means were compared using Tukey’s Honestly significant difference test [28].

**Table 1:** The primers used for Quantitative Real-time PCR.

Name of the target gene	Cluster ID	Forward Primer (5′-3′)	Reverse Primer (5′-3′)
Apolipophorin-III isoform 2	CFSW289	AGTCCATCCACTGACAACCA	CAGCGTTAGAGACAGCTTGC
Asparaginyl endopeptidase-like cysteine peptidase (AEP)	CFSW655	GCTGATAGCCTAGGTCAAGCTC	GAGCTCCATGAAGGAGGAAT
Calpain B	CFSW1228	AGGCGGAGTGGTAGAAAGAA	TACAACAAAGGGATGAGCCA
Carboxypeptidase b	CFSW30	GGAACTTCCACCAACGTTCT	ATATATGAGGCGGGCAACTC
Cathepsin D	CFSW621	CAACCCGACTATCACGTTTG	CTCAACTCCAACGTCCAAGA
Cathepsin L	CFSW1087	TGCTGGTCCTTCAGTGCTAC	TAAGCACACCCGTCTTTCTG
Cathepsin O	CFSW285	AGTGTCCAGGAGGTCAGGTC	ACATCTTCTGCACCAACCAA
Cysteine-rich protein 1 (CRP1)	CFSW799	CCTGCTACTCCGCACTCTT	CAACACAGGTTACAAACGCA
endo-β-1,4-glucanase (GH9)	CFSW14	CCAGTATGCCAAGAAGCAGA	ACGTGTTCCAGTCACATGCT
Ferritin 2	CFSWB75	GGCTACCATGATCCAGAGGT	ATGTAACCAGCCAACTCACG
Ferritin light chain	CFSW6	GGCTACCATGATCCAGAGGT	CGTGTAACCAGCCAACTCAC
Four-and-a-half LIM domain protein	CFSWB11	TGCATTTGACACAGCGAGTA	CATAGGCACCAAGAGCTTCA
Gram-negative bacteria-binding protein (GNBP1)	CFSWEC142	TCGTCAGCGAAACTATGACC	GGGTAACACTTTGGTGGCTT
Gram-negative bacteria-binding protein 2 (GH16)	CFSW160	ACAATCCCTGGGAATATGGA	TCACTCCTCCAACAGCTACG
Kazal-type serine protease inhibitor	CFSW43	TGGGACAGACAGGACAACAT	TCATTCTTCACAAGGACCCA
Hemolymph lipopolysaccharide binding protein	CFSW562	GGTTTACGTCGGTGTCAGTG	GTGGAACCAGATCGGAAAGT
Lysosomal Pro-X carboxypeptidase	CFSW1001	ATTCTGGTCCTCTGCGTCTT	GTCGGACGTGTATTTGATCG
Lysozyme-1 (c-type)	CFSW195	TCGGTTGTTCTCTGCTGTTC	AGGACAGCAATGTGCAGAAG
Lysozyme (i-type)	CFSW1341	GGGCCCAGTGAGTGAACTAT	GATCGATTGCAGTTGGACAC
Lysozyme (p-type)	CFSW1263	TAGGTGCCGGTCCTTGTAAT	ATAAACAGAGAGACCGCGTTT
Metacaspase-like cysteine peptidase (C14 family, Clan CD)	CFSW1304	TAGGTGCCGGTCCTTGTAAT	ATAAACAGAGAGACCGCGTTT
Prolixicin antimicrobial peptide	CFSW1405	TGGTACGGTGGTGAGAGGTA	CTGGACCGTTCAACACTCTG
Prophenoloxidase activating factor	CFSW1094	GTCATTGGCACCAGTTATGC	ACTGAACCGATCACACCAAA
Serine protease	CFSW1504	AAGAAATCCATGCAGCACAG	TTTCGAGCAAACTCTGGTTG
Termicin	CFSW277	TTGTCTTTCTGGTCGCAGTC	GTAGATGCTGTGCTGTGCCT
Thaumatin-like protein	CFSW23	AGATGAGACCAACACGTGGA	CATCCTGGGTTCAGATTCCT
Thaumatin-like protein	CFSW65	AGCGGGTTGTAGAAATGACC	TGGTAGCTGCACAGGAACTC
Transferrin	CFSWM110	GACAGAGTGGATTGCTTGGA	TCCTTGGTTCGAACTTCCTT
14-3-3 protein 1	CFSWM165	TGGTGCAAGAAGAAATGGTC	ATCCTTCCAATTCACCAAGC
β-actin	*	AGCGGGAAATCGTGCGTGAC	CAATAGTGATGACCTGGCCGT

* Zhou et al. [90].

## Results and Discussion

### 3.1 Characterization of the Normalized cDNA Library

The analysis of 3,000 randomly selected white clones from the normalized *C. formosanus* Shiraki cDNA library resulted in 2,788 high quality and trimmed sequences. After computationally clustering and assembling the sequences, a total of 1,511 non-redundant sequences (clusters) were obtained (Table 2). This included 1,149 singletons containing only one expressed sequence, and 362 multi-member expressed sequence clusters (contigs) ranging from 2 to 118 members. Most of the 315 multi-member clusters (87.02%) contained fewer than six expressed sequences. Only 5.52% of the clusters contained more than 10 sequences, and 2.49% included more than 20 sequences (Table S1). These results revealed excellent normalization of the cDNA library.

**Table 2:** Summary statistics of expressed sequence analyses from the full-length normalized cDNA library of immunized *C. formosanus* workers.

cDNA library characteristics	Number
Titer of cDNA library (cfu ml^−1^)	2.1 × 10^6^
Total cDNA clones picked and sequenced	3,000
Gene discovery rate*	55.53%
Sequences passing quality check	2,788
Total number of clusters	1,511
Singletons	1,149
Contigs	362

* Gene discovery rate was calculated as the total number of clusters divided by total number of sequences passing the quality check.

**Figure 1:**
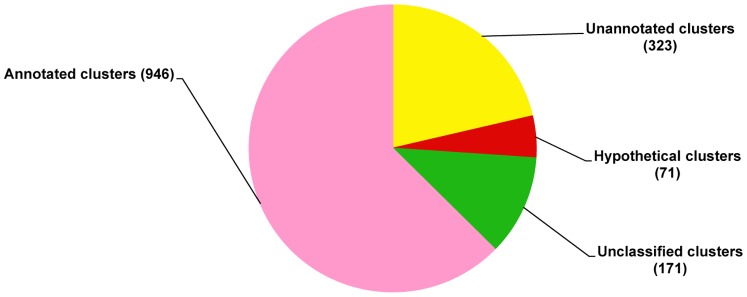
Distribution of 1,511 *C. formosanus* Shiraki clusters arranged by similarity to known genes as determined by Blast2Go annotation criteria. Footnote. Annotated clusters share similarity with annotated sequences in public databases. Unclassified clusters have no sequence similarity with any sequence in public databases. Hypothetical clusters share similarity with proteins of unknown function in public databases. Unannotated clusters share low (below cutoff E-value >10^−5^) similarity with sequences of unknown functions in public databases.

**Figure 2:**
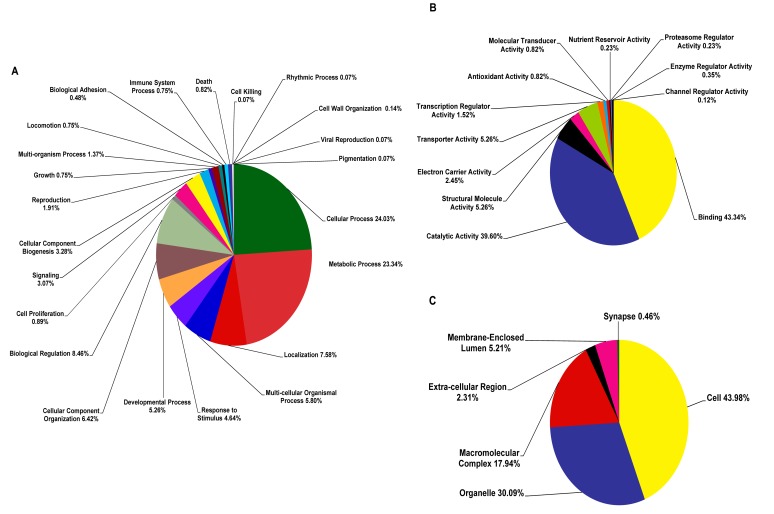
Categorization of 942 annotated clusters of *C. formosanus* Shiraki in Gene Ontology (GO) terms (level two) for (A) biological process, (B) molecular function, and (C) cellular component with a filter score cutoff E-value of 10^−5^.

Based on blastx analysis, the greatest percentage of the clusters (62.61%) exhibited significant similarity to known genes in the non-redundant database (expected value ≤10^−5^) as shown in the annotated clusters in Figure 1. The 71 hypothetical clusters accounted for 4.70% of non-redundant sequences. The remaining clusters were either unannotated as 21.38% (below the cutoff E-value) or unclassified as 11.32% (no sequence similarity to any sequence in public databases), suggesting that a significant number of clusters assembled in the normalized cDNA library of *C. formosanus* workers are novel (Figure 1). The annotated clusters were classified into functional categories including molecular functions, cellular components and biological processes. The proportion of clusters falling into each functional category is described in Figure 2. The expressed sequences have been submitted to the National Center for Biotechnology Information (GenBank accession number: JZ107278- JZ110065).

### 3.2 Characterization of the Suppression Subtractive Hybridization Libraries

Analysis of 800 randomly selected white clones from the four SSH libraries of *M. anisopliae*, *B. bassiana*, *B. thuringiensis* and *E. coli* -infected workers resulted in 88%, 89%, 86% and 94% of high quality sequences, respectively. The high quality ESTs in each SSH library were assembled to produce a non-redundant sequence set. Each SSH library had 56–62% clusters with significant similarity to known genes in the non-redundant database (expected value ≤10^−5^). However, each SSH library contained 33–40% of clusters with no significant sequence similarity to any sequence in the non-redundant database. The gene discovery rate of the SSH library constructed from *C. formosanus* workers immunized with *M. anisopliae* was the highest (69.27%) and contained the greatest number of singletons among the SSH libraries (Table S2). The identified clusters in the SSH libraries exhibited the greatest homology with the genome of *C. formosanus* (Table S3). The expressed sequences from these SSH libraries were submitted to the National Center for Biotechnology Information to obtain GenBank accession numbers.

### 3.3 Immune-related Genes in *C. formosanus* Shiraki

**Figure 3:**
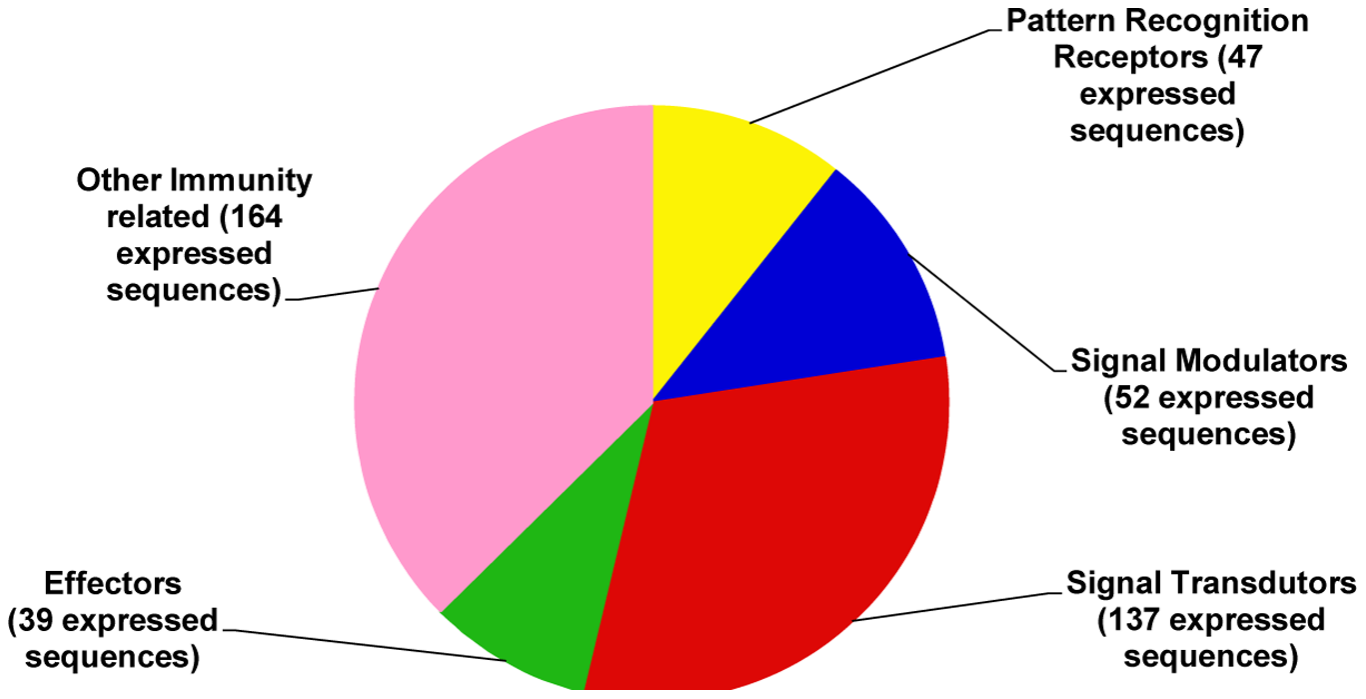
Categorization of immune-related expressed sequences of *C. formosanus* Shiraki with a filter score cutoff E-value of 10^−5^.

The expressed sequences generated from the full-length normalized cDNA library of Formosan subterranean termite workers infected with the studied microbes enabled us to explore immune function protein genes that termite workers may use in response to fungal and bacterial infection. To compile an immune response database for *C. formosanus* Shiraki, the sequences were mined and yielded 259 clusters that are involved in the humoral immune response (Table S4, Table S5, Table S6, Table S7 and Table S8). These clusters identified humoral response genes comprising genes for melanization, genes related to antimicrobial effector molecules, and genes involved in synthesis pathways of antimicrobial effector molecules. These immune-related clusters were then classified into five functional categories, such as pattern recognition receptors (PRRs), signal modulators, signal transductors, effectors and others (Figure 3).

From four SSH libraries, 81 immune-related clusters were identified. Further analysis revealed that 27, 17, 22 and 15 clusters are putatively involved in the immune response to infections with *M. anisopliae*, *B. bassiana*, *B. thuringiensis* and *E. coli*, respectively (Table 3). These genes encoded pattern recognition receptors, signal modulators, signal transductors, effectors and others, similar to that in the normalized cDNA library (Table S9).

**Table 3:** The number of identified immune-related genes in the SSH libraries.

Classification	SSH Libraries			
	*M. anisopliae*	*B. bassiana*	*B. thuringiensis*	*E. coli*
Pattern recognitionreceptors	2	1	2	2
Signal modulators	2	1	2	0
Signal transductors	9	3	9	4
Effectors	1	3	1	1
Other immunityrelated	13	9	8	8
Total	27	17	22	15

Among 259 identified immune-related clusters in the normalized cDNA library, 222 are unique to this library and 37 are also found in the SSH libraries, On the other hand, among 56 identified immune-related clusters from the SSH libraries, 19 are unique (Table 4). Normally, the immune-related clusters identified in the normalized cDNA library can also be identified in the SSH library if a sufficient number of clones were sequenced. However, the presence of 222 unique immune-related clusters in the normalized cDNA library revealed that the sequencing of 800 white clones from the four SSH libraries was not sufficient to cover all immune-related genes. Even so, the identification of immune-related clusters from each SSH library and unique clusters from four SSH libraries further enhances our understanding of the immune related transcriptome of *C. formosanus* Shiraki to various microbes. Obviously, the SSH libraries supplemented the discovery of immune-related genes together with the normalized cDNA library. Furthermore, the ESTs obtained from the SSH libraries showed higher gene discovery rate and low rebundancy (Table S2) compared with the previously published SSH library with 182 ESTs assembled into 19 clusters generated from *R. flavipes* in order to explore the immune related genes [18].

**Table 4:** Comparison of the number of identified immune-related clusters between the normalized cDNA library and the suppression subtractive hybridization (SSH) libraries.

	SSH Libraries*	Shared	Normalized cDNA library
Pattern recognition receptors	4	3	16
Signal modulators	3	2	31
Signal transductors	19	14	95
Effectors	4	3	19
Other immunity related	26	15	98
Total	56	37	259

* The same cluster present in more than one SSH library is counted as a single cluster.

#### 3.3.1 Microbial recognition

Immune reactions are initiated after successful recognition of potential pathogens. Microbial recognition is supposed to occur through conserved pathogen-associated molecular patterns (PAMPs) that are absent in the host. These PAMPs include β-1,3-glucans from fungi and lipopolysaccharides (LPSs) or peptidoglycans (PGs) from bacteria [29]. These PAMPs, after recognition, bind to host proteins, which are generally known as pattern recognition receptors (PRRs). The genome of invertebrates includes more than 10 groups of PRRs, including β-1,3-glucan recognition proteins (βGRPs), C-type lectins (CTL), Down syndrome cell adhesion molecules (DSCAM), fibrinogen-like domain immunolectins (FBNs), galectins (GALE), gram-negative binding proteins (GNBPs), hemolin, peptidoglycan recognition proteins (PGRPs), multidomain scavenger receptors (SCRs), nimrods, and thioester-containing proteins (TEPs) [29]–[30]. The exact mechanism by which Formosan subterranean termites, *C. formosanus* Shiraki detect infectious microorganisms is largely unknown. The current view is based on a homology search of our expressed sequences with known recognition-related genes in other insect species. Our database revealed that 47 expressed sequences from the normalized cDNA library are potential PRRs (Figure 3). These sequences were identified as forming 16 clusters encoding 10 types of proteins including β-1,3(4)-glucanase LIC1 (GH16), Gram-negative bacteria binding protein 2 (GH16), β-glucosidase (GH1), endo-1,3- β-glucanase (GH2), endo-β-1,4-glucanase (GH9), C-type lectin (CTL), Apolipophorins, Hemolymph lipopolysaccharide-binding protein, Immunoglobulin I-set domain -containing protein and Scavenger receptor class C (Table S4). Four genes for PRRs are represented in the SSH libraries, three of which are identical with those from the normalized cDNA library; only the Gram-negative bacteria binding protein 1 (GNBP1) is uniquely found in the SSH library generated from the infected *C. formosanus* workers with *E. coli* (Tables S9 and 4).

Four different families of glycosyl hydrolases (GHs) such as GH1, GH2, GH9 and GH16 were found in the normalized cDNA and SSH libraries of immunized *C. formosanus* Shiraki workers (Tables S4 and S9). Currently, only the members of the GH16 family have been reported as PRRs that play a role in the innate immune system of insects. Genes encoding Gram-negative binding proteins (GNBPs, GH16) and β-1,3-glucanase-like proteins (GH16) have been isolated from insects [31]–[36] and other invertebrates [37]. These proteins are involved in the recognition of microbial cell wall components such as β-1-3 glucan and presumably have two distinct glucan-binding domains, an N-terminal glucan-binding domain and a C-terminal domain that is similar to β-1,3- and β-1,4-glucanase-like domains [34]–[35], [38]. In termites, GNBPs are believed to act as antimicrobial effector proteins along with their basic function as immune elicitors [31]. In addition, the comparative molecular evolution analysis of GNBPs with another immune related Relish gene from 13 Australian termite species (*Nasutitermes*) revealed that GNBPs have experienced relatively less pressure to change the amino acid composition compared with Relish [14]. Recently, higher expression of gene coding for GNBP2 was observed among queens of *C. formosanus* compared to virgins [39]. In *Drosophila*, GNBP1 and GNBP3 are involved in the Toll pathway in response to Gram-positive bacterial and yeast infections, respectively [40]. However, according to our qPCR results, the gene encoding GNBP1 was up-regulated upon infection with *M. anisopliae*, *B. bassiana* and the Gram-negative *E. coli* but not with the Gram-positive *B. thuringiensis*. However, GNBP2 was also up-regulated on infection with Gram-negative *E. coli* (Table 5). This finding suggests that the activation of immune pathways in *C. formosanus* might be different from that in *Drosophila*. In some other termite species, such as *R. flavipes* and *R. virginicus*, GNBPs directly damage invading fungal pathogens through their β-1,3-glucanase activity [41]. Based on this evidence, they further suggested that these proteins are constitutively expressed and maintained on the termite cuticle and, in the nest, appear to be essential in preventing disease epizootics within the colonies by breaking down entomopathogenic fungi externally [41]. A GH9 endo-β-1,4-glucanase, which was found in our libraries, displayed a very special expression pattern; 11 sequences are found in the normalized cDNA library (Table S4). Some of the endo-β-1,4-glucanases showed close resemblance with already described same unigenes among *C. formosanus* Shiraki [42]–[43]. This protein was also identified in each of the four SSH libraries (Table S9). According to the q-PCR results, the relative fold expressions of these genes were extraordinarily high in every treatment: 14-fold in the treatment with the Gram-positive *B. thuringiensis*, 10-fold in the treatment with the Gram-negative *E. coli*, 9-fold in the treatment with *M. anisopliae* and 6-fold in the treatment with *B. bassiana* (Table 5) relative to control. Such a highly nonspecific up-regulated profile revealed that endo-β-1,4-glucanase in *C. formosanus* workers appears to act as like those of GNBPs in other termites to damage invading fungal pathogens directly in addition to their role in microbial recognition. The member of another GH1 family, β-glucosidase, was also identified in the SSH library treated with the Gram-positive *B. thuringiensis* (Table S9); therefore, this finding might represent a response to the recognition of Gram-positive bacteria.

**Table 5:** Relative fold expressions of immune-related genes determined using quantitative real-time PCR.

Protein name of the target Gene	Functional categories	*M. anisopliae*	*B. bassiana*	*B. thuringiensis*	*E. coli*
Hemolymph lipopolysaccharide-binding protein	PRR	1.46±0.03c	2.38±0.14b	1.54±0.10c	4.76±0.19a
Gram-negative bacteria-binding protein 2 (GNBP2)	PRR	1.05±0.01b	1.24±0.04b	1.41±0.25b	4.51±0.23a
Gram-negative bacteria-binding protein 1 (GNBP1)	PRR	2.63±0.21a	1.98±0.36ab	0.99±0.13b	1.76±0.22ab
Endo-β-1,4-glucanase (GH9)	PRR	9.29±0.37b	6.45±0.43c	14.60±0.35a	10.79±0.31b
Apolipophorin-III isoform 2	PRR	1.76±0.17a	0.36±0.01b	1.54±0.04a	0.49±0.01b
Kazal-type serine protease inhibitor	SM	6.67±0.40a	3.40±0.52b	4.58±0.41b	3.97±0.02b
Prophenoloxidase activating factor	SM	3.46±0.47a	1.24±0.15b	4.60±0.39a	4.81±0.25a
Serine protease	SM	1.31±0.08b	0.78±0.04c	1.98±0.18a	0.43±0.01c
14-3-3 protein 1	ST	7.17±0.17a	2.12±0.69b	9.12±1.48a	7.65±1.24a
Calpain B	ST	0.83±0.03c	0.99±0.04b	0.72±0.01c	1.80±0.03a
Four-and-a-half LIM domain protein	ST	0.33±0.14a	1.07±0.54a	1.07±0.14a	0.57±0.11a
Asparaginyl endopeptidase-like cysteine peptidase (AEP)	E	0.54±0.03c	2.20±0.02a	1.07±0.06b	1.15±0.02b
Cathepsin O	E	1.60±0.08c	1.99±0.05b	1.78±0.04bc	2.86±0.06a
Cathepsin L	E	0.68±0.05b	0.85±0.13b	1.02±0.07b	1.72±0.03a
Cathepsin D	E	1.57±0.03a	1.78±0.11a	0.84±0.03b	0.97±0.04b
Carboxypeptidase b	E	0.60±0.03d	0.88±0.05c	1.14±0.08b	1.55±0.02a
Lysozyme-1 (c-type)	E	0.80±0.01c	1.78±0.05b	2.88±0.04a	2.82±0.19a
Lysozyme (p-type)	E	1.17±0.04c	2.06±0.09b	2.73±0.16a	1.56±0.02c
Lysozyme (i-type)	E	0.84±0.03d	4.64±0.06a	3.04±0.07b	1.77±0.09c
Lysosomal Pro-X carboxypeptidase	E	1.59±0.15b	2.08±0.22b	2.85±0.13a	1.78±0.13b
Metacaspase-like cysteine peptidase	E	2.41±0.07c	3.71±0.31b	8.08±0.17a	7.62±0.13a
Prolixicin antimicrobial peptide	E	4.28±0.18a	2.77±0.12b	1.55±0.08c	0.44±0.02d
Termicin	E	3.74±0.21a	2.17±0.12bc	2.47±0.34b	1.38±0.03c
Thaumatin-like protein (Cluster ID CFSW65)	E	2.93±0.11b	1.08±0.03c	0.25±0.01c	4.36±0.38a
Thaumatin-like protein (Cluster ID CFSW23)	E	2.19±0.13b	0.81±0.05c	0.25±0.01d	3.67±0.06a
Cysteine-rich protein 1 (CRP1)	O	0.80±0.02c	1.12±0.04bc	2.02±0.05a	1.34±0.17b
Ferritin 2	O	4.99±0.54a	5.66±0.60a	1.68±0.43b	4.37±0.63a
Ferritin light chain	O	1.86±0.04b	2.65±0.06a	1.38±0.05c	1.55±0.03c
Transferrin	O	2.14±0.05a	0.98±0.06bc	1.68±0.37ab	0.77±0.03c

Means ± SE values having the same letter(s) along the row are not significantly different (based on the Tukey’s Honestly Significant Difference test; P<0.05). PRR: pattern recognition receptor; SM: signal modulator; ST: signal transductor; E: effector; O: other immune-related gene.

C-type lectins (CTLs) form a large family of sugar-binding proteins that are involved in the innate immune response by recognizing polysaccharide chains on the surface of pathogens [44]–[45]. Since the first CTL was described in *Bovine conglutinin* in 1906, a number of CTLs have been described in both vertebrates and invertebrates [45]. To date, many CTLs have been reported in various insect species, including 34 from the *D. melanogaster* genome, 25 from *A. gambiae*, 21 from the *Bombyx* genome (BmCTL1–21), 10 from *A. mellifera*, 10 from *T. castaneum*, and five from aphids [11]–[12], [46]. Only one expressed sequence encoding a protein similar to CTLs was identified in our normalized cDNA library (Table S4).

Lipopolysaccharide-binding proteins (LPSBPs) form a group of recognition proteins that bind to lipopolysaccharides (LPSs) on the bacterial surface. These types of binding proteins have been reported to clear bacteria from the hemolymph of *Periplaneta americana* [47]. Our expressed sequence analysis reveals that two sequences of one cluster are putatively involved in LPS binding (Table S4). The gene encoding the hemolymph LPSBP was significantly up-regulated (4.76-fold) after challenging with *E. coli* relative to control (Table 5). This protein might mainly respond to Gram-negative bacterial infections.

The members of Class C of the multi-domain scavenger receptor (SCR) family are capable of recognizing infectious bacteria (including gram-positive and gram-negative bacteria) [48]. They recognize multiple ligands and remove apoptotic cells and bacteria [49]. Different types of SCRC have been reported in different insects, for instance, four types are found in *Drosophila* and one from the *Anopheles* and *Apis* genomes [11]. One cluster identified from the normalized cDNA library was similar to the type I of SCRC as shown in Table S4.

The apolipophorin III (apoLp-III) family of proteins is a family of exchangeable apolipoproteins with multiple functions. One important function involves insect innate immunity. These proteins bind to bacterial and fungal cell wall molecules, such as the LPSs of Gram-negative bacteria, the lipoteichoic acid (LTA) of Gram-positive bacteria and the β-1,3-glucans of fungi to inhibit the growth of bacteria and fungi in *Galleria mellonella* [50]–[51]. Four apoLp-III sequences and two apolipophorin sequences were detected in our normalized cDNA library (Table S4). ApoLp-III was also identified in the SSH library treated with the fungus *M. anisopliae* (Table S9). ApoLp-III isoform 2 was significantly up-regulated upon infection with the fungus *M. anisopliae* and the Gram-positive *B. thuringiensis* (Table 5). These results suggest that apoLp-IIIs are also involved in the recognition of fungi and Gram-positive bacteria in termites. This recognition receptor binds to pathogen-associated molecular patterns such as β-1,3-glucan and fungal cells, and detoxifies LPS [50].

Finally, one cluster from our initial *C. formosanus* Shiraki immune gene project exhibited weak similarity to immunoglobulin (Ig)-superfamily receptors. Some members of this superfamily have already been reported in other insects, including *D. melanogaster*, *A. gambiae*, *B. mori* and *A. mellifera* [11].

#### 3.3.2 Signal modulation

After recognition of PAMPs by pattern recognition receptors, signal modulation proteins, such as serine proteases (SPs) and serine protease inhibitors (SPIs), amplify pathogen invasion signals, thereby activating various lines of defense against the invading pathogen. Such molecules are involved in hemolymph coagulation, antimicrobial peptide synthesis and the activation of phenoloxidases (POs) in invertebrate defense [52]–[53]. Fifty-two of the expressed sequences in our normalized cDNA library and three genes in the SSH libraries are potentially involved in signal modulation (Tables S5 and S9).

We identified 20 sequences belonging to nine clusters that encode SPs from the normalized cDNA library (Table S5). A gene encoding a serine protease homolog (SPH) that lacks protease activity is also proposed to participate in the innate immune response of *B. mori* [54]. We identified a sequence that codes for SPH 42 isoform 1 in the normalized cDNA library (Table S5). A gene encoding a SP was also detected in SSH libraries treated with *M. anisopliae*, *B. bassiana* and *B. thuringiensis* (Table S9). This gene was up-regulated after infection with *M. anisopliae* and *B. thuringiensis* (Table 5). These results indicate that SPs are involved in the modulation of fungi and Gram-positive bacteria through the Toll signaling pathway [40].

In insects, more than 20 families of SPIs are known. The most important families are the Kazal, Kunitz, alpha-macroglobulin and serpin families [55]. SPIs function to prevent inappropriate activation of the immune response [40]. We have identified eight sequences belonging to four clusters encoding Kazal-type serine protease inhibitors and one encoding a Pacifastin-related peptide precursor in the normalized cDNA library (Table S5). The gene encoding the Kazal-type serine protease inhibitor was up-regulated 3.4–6.7 fold after fungal and bacterial infections relative to control (Table 5); this nonspecific up-regulation profile is similar to that of the gene encoding endo-β-1,4-glucanase (PRRs). The serpin is dominant in other insects; for example, the genomes of other insect species, such as *A. mellifera*, *A. gambiae, B. mori* and *D. melanogaster*, contain 5, 17, 26 and 30 serpin genes, respectively [11]. However, no expressed sequence in any library exhibited homology to the serpin in termites. To determine whether the serpin is absent in termites and whether SPIs take the role of serpins, further study is required.

The prophenoloxidase (proPO) system is an important part of host defense in insects. The enzymatically inactive form, proPO, is cleaved into active phenoloxidase (PO) by an SP, prophenoloxidase-activating enzyme (PPAE) [40]. Activation of the proPO cascade leads to melanization, resulting in the production of quinones. These highly reactive and toxic compounds cause the encapsulation of invading microbes, which ultimately allows insects to eliminate invaders [56]. We identified one sequence encoding prophenoloxidase-activating factor (proPOAF) in the normalized cDNA library. q-PCR results revealed that all of the microbes tested caused significant increases in proPOAF gene expression in *C. formosanus* Shiraki workers [1.24–3.46 -fold upon infection with fungi and 4.6–4.8 -fold upon infection with bacteria relative to control (Table 5)]. These results suggest that proPOAF is an important component of *C. formosanus* Shiraki innate immunity.

#### 3.3.3 Signal transduction

The immune response of insects against pathogens (fungi and bacteria) mainly relies on signaling pathways including the immune deficiency (IMD), Toll, c-jun N-terminal kinase (JNK), and the janus kinase/signal transduction and activator of transcription (JAK/STAT) pathways [10]. These signaling pathways induce the expression of effectors that have been well characterized in *Drosophila*. However, little is known about the signaling pathways in Formosan subterranean termites. Among the analyzed sequences from *C. formosanus* Shiraki, we found approximately 137 expressed sequences belonging to 95 signal transduction-related clusters in the normalized cDNA library (Table S6). We identified 19 signal transduction-related genes in the SSH libraries (Table S9).

Toll-like receptors (TLRs) in the Toll pathway play important roles in recognizing specific microbial components derived from Gram-positive bacteria, fungi, protozoa and viruses [57]. The nucleotide-binding leucine-rich repeat (NLR) and NACHT-domain (NAIP (neuronal apoptosis inhibitor protein), C2TA (MHC class 2 transcription activator), HET-E (incompatibility locus protein from *Podospora anserina*) and TP1 (telomerase-associated protein) containing proteins are important components in the innate immune signaling pathway [58]–[59]. Our database showed the presence of genes encoding components of the TLRs, such as NLR-containing protein family (eight sequences in five clusters) and the NACHT-domain (one sequence of NACHT and the WD40 domain protein) (Table S6).

Relish, which has an NH2-terminal Rel homology domain and a COOH-terminal ankyrin repeat domain similar to that of the NF-κB inhibitor IKB, is a transcription factor that directly activates antibacterial target genes in the IMD pathway of *Drosophila* [60]. Four sequences in two clusters encoding the ankyrin repeat protein were identified in the normalized cDNA library. Moreover, two sequences in a cluster encoding Caspase-1, an important component associated with Relish in the IMD pathway, was identified in the normalized cDNA library. One sequence encoding JNK stimulatory phosphatase (jsp-1) and five clusters encoding serine/threonine protein kinases in the normalized cDNA library (Table S6) revealed the involvement of JNK and the mitogen-activated protein kinase kinase kinase (MAPKKK) cascade in this termite.

Members of the Ras (Rat sarcoma) superfamily have been known to be involved in a variety of signal transduction pathways that regulate the expression of genes for cell proliferation, differentiation, survival and apoptosis [61]. These proteins are divided into six major families including Ras, Rab, Rho, Ran, Arf and Rheb [61]–[62] and have recently been reported to play roles in the cellular immune response of *Spodoptera exigua* [63]. Some members of this superfamily have already been annotated in some other insect species, including *Drosophila*, to be involved in complex multiple signaling pathways [64]. Our sequence analysis revealed that 23 sequences from the normalized cDNA library belong to the Ras superfamily including the Ras, Rab and Rho families (Table S6). Sequences encoding the Rab GDP-dissociation inhibitor were identified in the SSH libraries obtained after treatment with fungi and Gram-negative bacteria (Table S9).

The members of the EF hand domain family play a pivotal role in calcium regulation within cells and in other physiological processes, including the immune defense mechanism that is activated upon exposure to external stimuli such as pathogens, light and stress [65]. Twenty-two sequences that belong to 14 clusters exhibited homology to EF-hand domain -containing proteins (Table S6). Genes encoding EF hand family proteins are also detected in the SSH libraries obtained after treatment with fungi and Gram-positive bacteria (Table S9). A gene encoding Capain B, which includes EF hand motifs in the Ca^2+^-binding domain [66], was up-regulated significantly upon infection with Gram-negative bacteria (Table 5). These results reveal that EF hand family proteins are important for the termite defense against fungi and bacteria.

In *Drosophila*, it has been reported that 14-3-3 proteins are mediators of bacterial phagocytosis and essential for normal immune defense [67]. Their role in signal transduction in plants and insects is well established [67]–[68]. Microbial challenge to *C. formosanus* Shiraki also induced the expression of nine sequences in four clusters encoding 14-3-3 proteins (Table S6). These sequences were also identified in the SSH libraries treated with *M. anisopliae* and *B. thuringiensis* (Table S9). These sequences were upregulated 7–9 -fold upon infection with bacteria and *M. anisopliae* and by 2-fold upon infection with *B. bassiana* relative to control (Table 5). These results suggest that these sequences may play important roles in termite defense.

In addition to the immune signaling proteins mentioned above, we identified genes encoding calreticulin, Sel-1, and other signal transduction related proteins, such as α-tubulin, ejaculatory bulb-specific protein, and phosphoenolpyruvate carboxykinase (PEPCK) in the normalized cDNA and SSH libraries. In *Drosophila*, calreticulin is an important marker for phagocytosis on the surface of apoptotic cells for targeting by the low-density lipoprotein receptor-related protein (LRP) [69]. Three sequences in two clusters encoding members of the calreticulin family were identified in the normalized cDNA library (Table S6). Members of the Sel1-like repeat (SLR) family mediate interactions between bacterial and eukaryotic host cells [70]. Six sequences in five clusters encoding SLR proteins were found in the normalized cDNA library (Table S6). The discovery of these genes would improve our understanding of the immune response mechanism against pathogens at the molecular level among *C. formosanus* Shiraki.

#### 3.3.4 Putative antimicrobial peptide diversity

Insect antimicrobial peptides (AMPs) and proteins are the final effectors that insects produce upon microbial infection. AMPs from model insects, such as *D. melanogaster*, *B. mori* and *T. molitor*, have been well studied [40], [71]–[72]. It is known that Defensin, Drosomycin, Cecropin, Metchnikowin, Tenascin 1 and Tenascin 2 are AMPs that act through the Toll pathway against fungi and Gram-positive bacteria [40], [72]–[74]. Diptericin, Drosocin, Cecropins, Attacins, Gloverin, Enbocin, Moricin, Lebocin and Tenascin 2 are products that act through the IMD pathway against Gram-negative bacteria [71]–[72], [75]–[76]. The normalized cDNA library analysis of *C. formosanus* Shiraki identified several expressed sequences that encode for antimicrobial peptides and proteins. Termicin, prolixicin, thaumatin-like proteins, cathepsins and lysozymes were prominent among these sequences (Table S7). A gene encoding prolixicin was first identified from our *C. formosanus* Shiraki transcriptome. Prolixicin might be related to the diptericin/attacin family [77]. Previously, this protein has been characterized in *Rhodnius prolixus* (Hemipteran) and exhibited strong activity against *E. coli*. Furthermore, the differential response of recombinant prolixicin has also been reported against several Gram-positive and Gram-negative bacteria [77]. Our qPCR results demonstrated 4.28 and 2.77-fold increases in Prolixicin expression from worker termites infected with the fungi *M. anisopliae* and *B. bassiana* and 1.55-fold with the Gram-positive *B. thuringiensis* relative to control. However, the expression of Prolixicin was down-regulated in the presence of *E. coli* (Table 5). These results indicate that Prolixicin is most likely produced through Toll pathway responses to fungi and Gram-positive bacteria. In addition, six expressed sequences exhibited homology with a previously described, cysteine-rich, defensin-like isopteran antimicrobial peptide, termicin (Table S7). This peptide, which exhibits strong antifungal activity, was first isolated from the fungus-growing termite, *Pseudacanthotermes spiniger* [78]. Recently, Xu et al. [79] reported 94 and characterized 67 new termicin mRNA sequences from two termite species including *Reticulitermes chinensis* (21) and *Odontotermes formosanus* (46). Termicin and GNBP2 knockdown decrease external cuticular antifungal activity in *R. flavipes*; therefore, termicins and GNBPs appear to provide multifaceted protection as internal effectors and receptors and as external effectors and sensors [80]. A quantitative expression analysis based on our qPCR results revealed significant up-regulation of termicin among worker termites upon exposure to all studied fungi and bacteria. Termicin was 2.17–3.74 -fold up-regulated in the presence of Toll pathway-related fungi and Gram-positive bacteria and 1.38-fold with IMD pathway-related Gram-negative bacteria relative to control (Table 5). These results suggest that termicins produced in *C. formosanus* not only respond to fungal and Gram-positive bacteria but also to Gram-negative bacteria.

Eight sequences belonging to three clusters encoding thaumatin-like proteins (TLPs) were identified in the normalized cDNA library (Table S7). These sequences were also found in SSH libraries obtained after treatment with *M. anisopliae* and *E. coli* (Table S9). The quantitative expression of these sequences revealed significant up-regulation among workers infected with *M. anisopliae* and *E. coli*. The qPCR results revealed 3.67–4.36 -fold upregulation upon *E. coli* infection and 2.19–2.93 -fold upregulation upon *M. anisopliae* infection relative to control. However, these sequences were significantly down-regulated upon infection with the Gram-positive *B. thuringiensis* (Table 5). These sequences code for disulfide-bridged polypeptides containing approximately 200 residues. TLPs have long been known to be synthesized in plants in response to stress, infection and developmental signals [81], and have also been reported to inhibit the conidial germination in *B. bassiana* and *Fusarium culmorum* [82]. Among insects, TLPs have been reported from the genomes of *Acyrthosiphon pisum* and *T. castaneum* [46]. However, the genome of the model insect *D. melanogaster* and some other insect species, such as *A. mellifera, A. gambiae, Pediculus humanus* and *Ixodes scapularis,* lacked the transcripts for thaumatin-like proteins [46]. The presence of TLPs in the genome of only a few insect species might suggest that these insects acquired these genes independently.

Five sequences in three clusters encoding c-type lysozymes, one sequence of an i-type lysozyme and one sequence of a protist-type lysozyme were also observed among the sequences of the normalized cDNA library (Table S7). Lysozymes are widely distributed in nature. Thus far, seven types of lysozymes have been found in various organisms: c-type lysozyme from chicken, g-type lysozyme from goose, i-type lysozyme from invertebrates, protist-type lysozyme from nematode, plant-type lysozyme from plants, bacteria-type lysozyme from bacteria and phage-type lysozyme from bacteriophage T4 [83]–[84]. Eight isoforms of c-type lysozyme in *D. melanogaster* mainly act in digestion, whereas a single copy of immune-related c-type lysozyme is present in Lepidopteran insects [85]. Three isoforms of the i-type lysozyme were also identified in *D. melanogaster*. However, the protist-type lysozyme found in our normalized termite cDNA library is the first evidence for the presence of a protist-type lysozyme in insects. Lysozymes are secreted by salivary glands and spread by allogrooming, an activity through which termite workers feed their nestmates [86]. These proteins are known to function as digestive enzymes in *Hodotermopsis sjostedti* and egg recognition pheromones in *Reticulitermes speratus,* and may act as anti-bacterial agents in both cases [87]. According to the qPCR results, three types of lysozyme in *C. formosanus* exhibited different expression patterns. Approximately 2.8-fold up-regulation of c-type lysozyme was observed upon infection with bacteria relative to control. The i-type lysozyme was highly expressed among workers infected with *B. bassiana* and *B. thuringiensis*. An increasing trend of protist-type lysozyme expression was also found with *B. bassiana*, *B. thuringiensis* and *E. coli* (Table 5). Further studies are needed to uncover the functions of these types of lysozyme and how they operate within the termite immune system.

Several sequences exhibited homology with genes encoding Cathepsin D, L, O and Asparaginyl endopeptidase-like cysteine peptidase (AEP) in our normalized cDNA library (Table S7). The gene coding for Cathepsin D was up-regulated mainly upon infection with fungi. Our findings are in line with Hamilton that identified cathepsin D to be involved in antimicrobial activity among *Camponotus pennsylvanicus* nest-mates [88]. The expression of Cathepsin L increased upon infection with Gram-negative *E. coli,* and Cathepsin O responded to all tested microbes (Table 5). We also identified three isoforms of Metacaspase-like cysteine peptidases (the C14 family, or Clan CD) (Table S7). Genes encoding metacaspase-like cysteine peptidases were 7.6–8 -fold up-regulated upon exposure to bacterial suspensions and 2.4–3.7 -folds upon exposure to fungi relative to control (Table 5). These types of effectors have been characterized in several insects and are believed to play a pivotal role against microbial infection [89].

### 3.4 Conclusion

Understanding the defense and innate immune mechanisms in termites is very important for developing sustainable control strategies. Based on our immune normalized cDNA library, SSH libraries and qPCR results, we characterized genes encoding pattern recognition receptors (17 clusters), signal modulators (32 clusters), signal transductors (100 clusters) and antimicrobial peptides (20 clusters). These methods are found to be reliable for the identification and validation of immune related genes.

The nonspecific, highly up-regulated profile of the gene encoding endo-1,4-glucanase (GH9) revealed a potential, direct microbe-damaging function of endo-1,4-glucanase in addition to its role as a pattern recognition receptor in *C. formosanus* workers. The observed up-regulation of the gene encoding GNBP1 and GNBP2 upon infection with fungi and Gram-negative bacteria but not Gram-positive bacteria suggested that the immune pathway is potentially regulated differently in *C. formosanus* than in *Drosophila* and other insects. The identification of sequences encoding molecules that are involved in the Toll, IMD, JNK and other pathways, such as Toll-like receptors, Caspase-1, ankyrin repeat protein, Ras (Rat sarcoma) superfamily, EF hand domain, and 14-3-3 proteins, suggested that these conservative signal pathways are also important for innate immunity in *C. formosanus*. These regulatory pathways ultimately led the synthesis of unique Prolixicin antimicrobial peptides along with Cathepsins, Termicin, Thaumatin-like proteins, and c-type, i-type and protist-type lysozymes.

The current results lay the initial ground for the identification of genetic variations among immune related genes and their involvement in disease resistance of *C. formosanus* Shiraki to obtain better understanding of expression mechanism. More recently, the highly sophisticated methodologies regarding gene disruption are becoming available. Our findings might help to pave the way in future for the development of RNA interference mediated approach to control the colonies of termites.

## Supporting Information

Table S1 **Highly expressed sequences identified from the normalized cDNA library of** ***C. formosanus*** **Shiraki based on sequence similarity.**
(DOC)

Table S2 **Summary statistics of expressed sequence tags (EST) analyses from the SSH libraries of** ***C. formosanus*** **workers.** T_1_ represents *M. anisopliae*, T_2_ represents *B. bassiana*, T_3_ represents *B. thuringiensis*, T_4_ represents *E. coli.* * Gene discovery rate was calculated as the total number of clusters divided by the total number of sequences passing the quality check.
(DOC)

Table S3 **Species distribution of top hits for clusters from whole body** ***C. formosanus*** **workers immunized with different microbes (Blast×, cutoff ≤1e^−05^).**
(DOC)

Table S4 **Immune-related pattern recognition receptors identified from the full-length normalized cDNA library of immunized** ***C. formosanus*** **Shiraki based on sequence similarity (** ***E*** **≤10^−5^).**
(DOC)

Table S5 **Immune-related signal modulators identified from the full-length normalization cDNA library of immunized** ***C. formosanus*** **Shiraki based on sequence similarity (** ***E*** **≤10^−5^).**
(DOC)

Table S6 **Immune-related signal transductors identified from the full-length normalization cDNA library of immunized** ***C. formosanus*** **Shiraki based on sequence similarity (** ***E*** **≤10^−5^).**
(DOC)

Table S7 **Immune-related effectors identified from the full-length normalized cDNA library of immunized** ***C. formosanus*** **Shiraki based on sequence similarity (** ***E*** **≤10^−5^).**
(DOC)

Table S8 **Other immune-related expressed sequences identified from the cDNA library of** ***C. formosanus*** **Shiraki based on sequence similarity (** ***E*** **≤10^−5^).**
(DOC)

Table S9 **Identified putative immune-related genes from the Suppression Subtractive Hybridization (SSH) libraries of immunized** ***C. formosanus*** **Shiraki prepared using four microbe types.** Footnote of Table S9. - represents absent;+represents present; * indicates that the same gene has also been identified in the normalized cDNA library; ** indicates genes that were quantified using quantitative real time PCR (qRT -PCR). ST: signal transductor; SM: signal modulator; PRR: pattern recognition receptor; O: other immune-related; E: effector.
(DOC)
