# Supplementary material for: Immune-Related Transcriptome of Coptotermes formosanus Shiraki Workers: The Defense Mechanism
Source: PLoS One. 2013 Jul 16;8(7):e69543. doi: 10.1371/journal.pone.0069543 (PMC3712931; doi:10.1371/journal.pone.0069543)
Supplement: Table S1 — Highly expressed sequences identified from the normalized cDNA library of C. formosanus Shiraki based on sequence similarity. (DOC) [file pone.0069543.s001.doc]

**Table S1. Highly expressed sequences identified from the normalized cDNA library of *C. formosanus*** Shiraki based on sequence similarity

| **Cluster ID** | **No. of ESTs** | **Annotation** | **E-value** |
| --- | --- | --- | --- |
| CFSW1479 | 118 | Hypothetical protein | 1.00E-08 |
| CFSW1395 | 55 | ---NA--- | - |
| CFSW1 | 43 | Hypothetical protein | 3.2E-9 |
| CFSW450 | 46 | Actin | 1.6E-154 |
| CFSW3 | 37 | Aytochrome c oxidase subunit III | 1.9E-97 |
| CFSW5 | 33 | Glycosyl hydrolase family5 | 6.5E-68 |
| CFSW2 | 31 | Cytochrome c oxidase subunit I | 4.6E-106 |
| CFSW144 | 23 | jonah 65aiv | 7.9E-37 |
| CFSW6 | 23 | Ferritin light chain | 4.0E-65 |
