# Supplementary material for: Immune-Related Transcriptome of Coptotermes formosanus Shiraki Workers: The Defense Mechanism
Source: PLoS One. 2013 Jul 16;8(7):e69543. doi: 10.1371/journal.pone.0069543 (PMC3712931; doi:10.1371/journal.pone.0069543)
Supplement: Table S2 — Summary statistics of expressed sequence tags (EST) analyses from the SSH libraries of C. formosanus workers. T1 represents M. anisopliae, T2 represents B. bassiana, T3 represents B. thuringiensis, T4 represents E. coli. * Gene discovery rate was calculated as the total number of clusters divided by the total number of sequences passing the quality check. (DOC) [file pone.0069543.s002.doc]

**Table S2. Summary statistics of expressed sequence tags (EST) analyses from the SSH libraries of *C. formosanus*** workers.

|  | **Treatments** | | | |
| --- | --- | --- | --- | --- |
| **cDNA library characteristics** | **T_1_** | **T_2_** | **T_3_** | **T_4_** |
| Total cDNA clones picked and sequenced | 200 | 200 | 200 | 200 |
| Gene discovery rate* | 69.27% | 53.88% | 59.43% | 55.32% |
| Sequences passing quality check | 179 | 180 | 175 | 188 |
| Putative identified clusters | 70 | 58 | 60 | 64 |
| Hypothetical clusters | 5 | 5 | 3 | 5 |
| Unknown clusters | 49 | 34 | 41 | 35 |
| Total number of clusters | 124 | 97 | 104 | 104 |
| Singletons | 95 | 69 | 76 | 76 |
| Contigs | 29 | 28 | 28 | 28 |

T_1_ represents *M. anisopliae*, T_2_ represents *B. bassiana*, T_3_ represents *B. thuringiensis*, T_4_ represents *E. coli*

* Gene discovery rate was calculated as the total number of clusters divided by the total number of sequences passing the quality check.
