# Supplementary material for: Immune-Related Transcriptome of Coptotermes formosanus Shiraki Workers: The Defense Mechanism
Source: PLoS One. 2013 Jul 16;8(7):e69543. doi: 10.1371/journal.pone.0069543 (PMC3712931; doi:10.1371/journal.pone.0069543)
Supplement: Table S3 — Species distribution of top hits for clusters from whole body C. formosanus workers immunized with different microbes (Blast×, cutoff ≤1e−05). (DOC) [file pone.0069543.s003.doc]

**Table S3. Species distribution of top hits for clusters from whole body *C. formosanus* workers immunized with different microbes (Blast×, cutoff ≤ 1e-05).**

|  | **Treatments** | | | |
| --- | --- | --- | --- | --- |
| **Species** | ***M. anisopliae*** | ***B. bassiana*** | ***B. thuringiensis*** | ***E. coli*** |
| *Coptotermes formosanus* | 22 | 9 | 14 | 12 |
| *Tribolium castaneum* | 1 | 6 | - | - |
| *Blattella germanica* | 3 | 2 | 1 | 2 |
| *Pseudotrichonympha grassii* | 4 | 2 | 3 | 1 |
| *Trichomonas vaginalis* | 5 | 5 | 5 | 2 |
| *Periplaneta americana* | - | - | 1 | 4 |
| *Bombus terrestris* | 1 | - | 1 | 1 |
| Aureococcus anophagefferens | - | 1 | 1 | - |
| *Camponotus floridanus* | 1 | - | - | 5 |
| *Anopheles culicifacies* | 1 | - | - | 1 |
| *Nilaparvata lugens* | 1 | - | - | - |
| *Reticulitermes flavipes* | 1 | - | 1 | - |
| *Verrucomicrobium spinosum* | 1 | - | - | - |
| *Mastotermes darwiniensis* | 1 | - | - | - |
| *Acromyrmex echinatior* | 1 | 3 | 1 | 1 |
| *Schistocerca gregaria* | 1 | 1 | - | - |
| *Coptotermes sjoestedti* | 1 | - | - | 1 |
| *Culex quinquefasciatus* | 1 | - | - | - |
| *Thermobia domestica* | 3 | 2 | 1 | 1 |
| *Picea sitchensis* | - | 2 | 1 | 2 |
| *Neospora caninum* | 1 | 4 | 1 | 2 |
| *Biston betularia* | 1 | - | - | - |
| *Pediculus humanus* | 1 | 2 | 3 | 1 |
| *Dermatophagoides pteronyssinus* | 1 | 1 | - | - |
| *Vicia faba* | 1 | - | - | - |
| *Branchiostoma lanceolatum* | 1 | - | - | - |
| *Nasonia vitripennis* | 3 | - | 1 | 3 |
| *Aedes aegypti* | - | - | 1 | 2 |
| *Ixodes scapularis* | 1 | - | - | 1 |
| *Harpegnathos saltator* | 1 | - | - | 1 |
| *Treponema primitia* | 1 | - | - | 2 |
| *Bombyx mori* | - | 1 | 1 | - |
| *Megachile rotundata* | 1 | 3 | 3 | 1 |
| *Apis mellifera* | 1 | 1 | - | - |
| *Arthroderma gypseum* | 1 | - | - | 1 |
| *Helicoverpa armigera* | 1 | - | - | 1 |
| *Histomonas meleagridis* | - | - | 2 | - |
| *Acyrthosiphon pisum* | - | 2 | - | - |
| *Laodelphax striatella* | - | - | 2 | - |
| *Ornithorhynchus anatinus* | - | - | 2 | - |
| *Hodotermopsis japonica* | - | - | - | 2 |
| *Cryptocercus punctulatus* | - | - | - | 2 |
| Others | 10 | 16 | 17 | 17 |
