# Supplementary material for: Immune-Related Transcriptome of Coptotermes formosanus Shiraki Workers: The Defense Mechanism
Source: PLoS One. 2013 Jul 16;8(7):e69543. doi: 10.1371/journal.pone.0069543 (PMC3712931; doi:10.1371/journal.pone.0069543)
Supplement: Table S4 — Immune-related pattern recognition receptors identified from the full-length normalized cDNA library of immunized C. formosanus Shiraki based on sequence similarity ( E ≤10−5). (DOC) [file pone.0069543.s004.doc]

**Table S4. Immune-related pattern recognition receptors identified from the full-length normalized cDNA library of immunized *C. formosanus* Shiraki based on sequence similarity (*E* ≤ 10^-5^).**

| **Cluster ID** | **No. of Sequences** | **Annotation** | ***E*-value** |
| --- | --- | --- | --- |
| CFSW19 | 8 | β-1,3(4)-glucanase LIC1 (GH16) | 4.60E-138 |
| CFSW 160 | 5 | Gram-negative bacteria-binding protein 2 (GH16) | 9.60E-149 |
| CFSW 1407 | 1 | Gram-negative bacteria binding protein 2 (GH16) | 1.30E-112 |
| CFSW1506 | 4 | β-glucosidase (GH1) | 1.30E-155 |
| CFSW1233 | 1 | β-glucosidase (GH1) | 9.3E-90 |
| CFSW170 | 2 | β-glucosidase (GH1) | 3.40E-151 |
| CFSW946 | 1 | β-glucosidase (GH1) | 1.6E-141 |
| CFSW464 | 2 | endo-1,3- β-glucanase (GH2) | 6.50E-28 |
| CFSW14 | 11 | endo-β-1,4-glucanase (GH9) | 4.9E-140 |
| CFSW483 | 1 | C-type lectin (CTL) | 4.9E-14 |
| CFSW1412 | 2 | Apolipophorins | 1.2E-20 |
| CFSW162 | 3 | Apolipophorin-III | 7.20E-15 |
| CFSW289 | 1 | Apolipophorin-III isoform 2 | 5.60E-15 |
| CFSW562 | 2 | Hemolymph lipopolysaccharide-binding protein | 4.40E-11 |
| CFSW1109 | 2 | Immunoglobulin I-set domain containing protein | 1.30E-09 |
| CFSW510 | 1 | Scavenger receptor class C, type I CG4099-PA | 1.40E-05 |
