# Supplementary material for: Immune-Related Transcriptome of Coptotermes formosanus Shiraki Workers: The Defense Mechanism
Source: PLoS One. 2013 Jul 16;8(7):e69543. doi: 10.1371/journal.pone.0069543 (PMC3712931; doi:10.1371/journal.pone.0069543)
Supplement: Table S5 — Immune-related signal modulators identified from the full-length normalization cDNA library of immunized C. formosanus Shiraki based on sequence similarity ( E ≤10−5). (DOC) [file pone.0069543.s005.doc]

**Table S5. Immune-related signal modulators identified from the full-length normalization cDNA library of immunized *C. formosanus* Shiraki based on sequence similarity (*E* ≤ 10^-5^**).

| **Cluster ID** | **No. of Sequences** | **Annotation** | ***E*-value** |
| --- | --- | --- | --- |
| CFSW1231 | 1 | Arginine/serine-rich 7 | 2E-48 |
| CFSW596 | 1 | Arginine/serine-rich 18 | 1.20E-21 |
| CFSW1213 | 1 | Arginine/serine-rich 17A | 6.50E-87 |
| CFSW341 | 1 | Arginine/serine-rich 17A | 6e-54 |
| CFSW1435 | 1 | Chymotrypsin-like protein | 9.6E-43 |
| CFSW552 | 1 | Kazal domain-containing peptide | 5.00E-06 |
| CFSW724 | 1 | Kazal-type serine protease inhibitor | 9.10E-06 |
| CFSW43 | 3 | Kazal-type serine protease inhibitor | 3E-10 |
| CFSW288 | 2 | Kazal-type serine protease inhibitor | 7.0E-12 |
| CFSW680 | 1 | Kazal-type serine protease inhibitor | 2.1E-6 |
| CFSW1373 | 1 | Pacifastin-related peptide precursor | 8.10E-29 |
| CFSW1094 | 1 | Prophenoloxidase activating factor | 1.6E-22 |
| CFSW455 | 3 | Reeler, the N-terminal domain of reelin | 6.8E-17 |
| CFSW1134 | 2 | Serine-arginine protein 55 | 1.70E-97 |
| CFSW391 | 2 | Serine arginine repetitive matrix protein 1 | 2.50E-11 |
| CFSW700 | 1 | Serine protease | 4E-32 |
| CFSW154 | 5 | Serine protease | 3.70E-50 |
| CFSW912 | 1 | Serine protease | 3E-27 |
| CFSW274 | 5 | Serine protease | 3e-94 |
| CFSW745 | 1 | Serine protease | 1E-80 |
| CFSW913 | 1 | Serine protease | 4e-120 |
| CFSW169 | 2 | Serine protease | 4.20E-08 |
| CFSW1504 | 1 | Serine protease | 2.2E-47 |
| CFSW148 | 3 | Serine protease | 1.90E-40 |
| CFSW1253 | 1 | Serine protease homolog 42 isoform 1 | 2E-60 |
| CFSW627 | 3 | Serine 3-dehydrogenase | 8.10E-55 |
| CFSW1301 | 1 | Serine/threonine protein kinase | 4.00E-07 |
| CFSW971 | 2 | Tetraspanin 18 | 1E-94 |
| CFSW1315 | 1 | Tetraspanin D107 | 7.1E-62 |
| CFSW1464 | 1 | Tetraspanin | 1.9E-11 |
| CFSW1428 | 1 | Trypsin-like serine protease | 1.9E-40 |
