# Supplementary material for: Immune-Related Transcriptome of Coptotermes formosanus Shiraki Workers: The Defense Mechanism
Source: PLoS One. 2013 Jul 16;8(7):e69543. doi: 10.1371/journal.pone.0069543 (PMC3712931; doi:10.1371/journal.pone.0069543)
Supplement: Table S6 — Immune-related signal transductors identified from the full-length normalization cDNA library of immunized C. formosanus Shiraki based on sequence similarity ( E ≤10−5). (DOC) [file pone.0069543.s006.doc]

**Table S6. Immune-related signal transductors identified from the full-length normalization cDNA library of immunized *C. formosanus* Shiraki based on sequence similarity (*E* ≤ 10^-5^**).

| **Cluster ID** | **No. of Sequences** | **Annotation** | ***E*-value** |
| --- | --- | --- | --- |
| CFSW71 | 2 | 14-3-3 protein 1 | 1.60E-98 |
| CFSW888 | 1 | 14-3-3 protein 2 | 3.60E-82 |
| CFSW281 | 3 | 14-3-3 protein 3 | 2.60E-80 |
| CFSW454 | 3 | 14-3-3 protein 4 | 4.00E-76 |
| CFSW732 | 2 | α-tubulin-1 | 2.1E-122 |
| CFSW1458 | 1 | α-tubulin-2 | 4.6E-142 |
| CFSW992 | 1 | β-tubulin-1 | 1.0E-178 |
| CFSW1472 | 1 | β-tubulin-2 | 5.00E-172 |
| CFSW300 | 4 | β-tubulin-3 | 2.0E-78 |
| CFSW301 | 4 | β-tubulin-4 | 5.0E-164 |
| CFSW1238 | 1 | Ankyrin repeat protein | 3.10E-42 |
| CFSW214 | 3 | Ankyrin repeat protein | 1.00E-15 |
| CFSW958 | 3 | Annexin 1 | 4E-105 |
| CFSW182 | 2 | C2 domain containing protein | 4.70E-09 |
| CFSW635 | 2 | C2 domain containing protein | 1.70E-19 |
| CFSW666 | 1 | C2 domain containing protein | 2.4E-102 |
| CFSW670 | 1 | C2 domain containing protein | 1.60E-20 |
| CFSW825 | 1 | C2 domain containing protein | 6.10E-23 |
| CFSW1193 | 1 | Calmodulin-like protein | 1.10E-67 |
| CFSW1386 | 1 | c-Jun protein | 1.4E-49 |
| CFSW1228 | 1 | Calpain B | 5.60E-22 |
| CFSW415 | 2 | Calreticulin family protein | 5.10E-20 |
| CFSW991 | 1 | Calreticulin isoform 1 | 1.2E-162 |
| CFSW229 | 1 | CaM kinase family protein kinase | 4.20E-54 |
| CFSW647 | 2 | CaM kinase family protein kinase | 1.3E-80 |
| CFSW466 | 2 | Caspase-1 | 1.50E-33 |
| CFSW233 | 1 | Cofilin tropomyosin-type actin-binding protein | 8.00E-31 |
| CFSW240 | 1 | Cofilin tropomyosin-type actin-binding protein | 4.80E-44 |
| CFSW1264 | 1 | Class B secretin-like G-protein coupled receptor | 1.5E-32 |
| CFSW79 | 1 | COP9 signalosome complex subunit 2 | 7.2E-10 |
| CFSW173 | 1 | EF hand domain (c-terminal) containing 2 | 2.0E-64 |
| CFSW1085 | 2 | EF hand domain (c-terminal) containing 2 | 8.8E-62 |
| CFSW518 | 1 | EF hand domain-containing protein cg10641 | 6.7E-28 |
| CFSW35 | 4 | EF hand family protein | 4.3E-33 |
| CFSW126 | 1 | EF hand family protein | 2.2E-26 |
| CFSW296 | 2 | EF hand family protein | 1.5E-35 |
| CFSW423 | 1 | EF hand family protein | 3.1E-19 |
| CFSW706 | 1 | EF hand family protein | 1.3E-18 |
| CFSW504 | 1 | EF hand family protein | 5.2E-14 |
| CFSW291 | 1 | EF hand family protein | 1.3E-48 |
| CFSW84 | 1 | EF hand family protein | 2.0E-35 |
| CFSW36 | 3 | EF hand family protein | 1.1E-58 |
| CFSW1030 | 1 | EF hand family protein | 2.0E-50 |
| CFSW1103 | 2 | EF (penta) protein with a long n-terminal hydrophobic domain | 1.8E-68 |
| CFSW905 | 1 | Ejaculatory bulb-specific protein III | 3.10E-41 |
| CFSW964 | 1 | Phosphoenolpyruvate carboxykinase (PEPCK) | 1.00E-39 |
| CFSW907 | 4 | Phosphoenolpyruvate carboxykinase (PEPCK) | 3.0E-132 |
| CFSW1302 | 1 | GTP-binding protein rheb-like protein | 2.70E-20 |
| CFSW684 | 1 | GTP-binding protein era homolog | 1.10E-40 |
| CFSW434 | 1 | Inositol hexaphosphate kinase (InsP6K) | 1.30E-35 |
| CFSW404 | 1 | jnk stimulatory phosphatase (jsp1) | 1.3E-81 |
| CFSW364 | 2 | Leucine rich repeat family | 3.70E-15 |
| CFSW730 | 2 | Leucine rich repeat family | 7.20E-06 |
| CFSW440 | 1 | Leucine rich repeat protein 1 | 7.30E-47 |
| CFSW747 | 1 | Leucine rich repeat family | 1.90E-28 |
| CFSW1120 | 2 | Leucine-rich repeat flightless-interacting | 3.30E-06 |
| CFSW611 | 1 | Member of ras oncogene family-like 4 | 1.10E-36 |
| CFSW600 | 1 | Member Ras oncogene family | 1.00E-31 |
| CFSW1419 | 1 | Member Ras oncogene family | 1.30E-57 |
| CFSW823 | 1 | Member Ras oncogene family | 3.10E-49 |
| CFSW494 | 1 | NACHT and WD40 domain protein | 2.20E-43 |
| CFSW546 | 1 | Nucleolin: isoform CRA_b | 1.1E-16 |
| CFSW1204 | 1 | Nuclear progesterone receptor | 7.2E-48 |
| CFSW657 | 1 | Ras family protein | 2.50E-38 |
| CFSW316 | 1 | Ras family protein | 9.60E-69 |
| CFSW701 | 1 | Ras family protein | 4.60E-51 |
| CFSW99 | 1 | Ras GTPase | 9.50E-54 |
| CFSW692 | 1 | Rab acceptor protein 1 | 5.50E-33 |
| CFSW966 | 1 | Rab-11A | 1.2E-144 |
| CFSW1110 | 2 | Rab GTPase family 6 | 2.20E-105 |
| CFSW1230 | 1 | Rab geranylgeranyl transferase alpha subunit | 6.20E-34 |
| CFSW54 | 1 | Rab GTPase | 1.30E-57 |
| CFSW866 | 2 | Rho GTPase | 2.10E-71 |
| CFSW699 | 3 | Rho family GTPase | 8.90E-63 |
| CFSW354 | 2 | RhoGEF domain containing protein | 1E-39 |
| CFSW1203 | 1 | Rho-related GTP-binding protein | 1.60E-71 |
| CFSW1357 | 1 | Rribosomal protein s6 | 5.20E-105 |
| CFSW1325 | 1 | Sel1 domain containing protein | 7.6E-32 |
| CFSW367 | 1 | Sel1 domain containing protein | 8.6E-9 |
| CFSW1016 | 1 | Sel1 domain containing protein | 6.7E-9 |
| CFSW1017 | 2 | Sel1 domain containing protein | 5.1E-12 |
| CFSW76 | 1 | Sel1 domain containing protein | 2.6E-5 |
| CFSW901 | 1 | Serine/Threonine protein phosphatase | 1.3E-55 |
| CFSW77 | 1 | Serine/Threonine protein phosphatase | 1.7E-39 |
| CFSW1301 | 1 | Serine/Threonine protein kinase | 4.00E-07 |
| CFSW622 | 1 | Serine/Threonine protein kinase | 1.1E-37 |
| CFSW1177 | 1 | Serine/Threonine-protein kinase | 5.5E-26 |
| CFSW1009 | 1 | Serine/Threonine protein kinase | 1.0E-47 |
| CFSW87 | 1 | Serine/Threonine protein kinase | 1.90E-54 |
| CFSW1402 | 1 | Serine/Threonine protein kinase | 1.40E-41 |
| CFSW751 | 1 | Small GTP-binding protein | 1.30E-60 |
| CFSW192 | 2 | Small GTP-binding protein | 8.80E-59 |
| CFSW1303 | 1 | Signal peptidase I (Spase I) | 1.4E-78 |
| CFSW1290 | 1 | Zinc finger protein | 3E-29 |
| CFSW418 | 1 | Zinc finger protein | 3.30E-52 |
