# Supplementary material for: Immune-Related Transcriptome of Coptotermes formosanus Shiraki Workers: The Defense Mechanism
Source: PLoS One. 2013 Jul 16;8(7):e69543. doi: 10.1371/journal.pone.0069543 (PMC3712931; doi:10.1371/journal.pone.0069543)
Supplement: Table S7 — Immune-related effectors identified from the full-length normalized cDNA library of immunized C. formosanus Shiraki based on sequence similarity ( E ≤10−5). (DOC) [file pone.0069543.s007.doc]

**Table S7. Immune-related effectors identified from the full-length normalized cDNA library of immunized *C. formosanus* Shiraki based on sequence similarity (*E* ≤ 10^-5^**).

| **Cluster ID** | **No. of Sequences** | **Annotation** | ***E*-value** |
| --- | --- | --- | --- |
| CFSW655 | 1 | Asparaginyl endopeptidase-like cysteine peptidase (C13 family), AEP | 2.90E-36 |
| CFSW285 | 2 | Cathepsin O | 4.90E-10 |
| CFSW621 | 1 | Cathepsin D | 5.70E-71 |
| CFSW1087 | 2 | Cathepsin L | 1.80E-117 |
| CFSW167 | 3 | Metacaspase-like cysteine peptidase (C14 family, Clan CD) | 1.90E-19 |
| CFSW1304 | 1 | Metacaspase-like cysteine peptidase (C14 family, Clan CD) | 1.70E-55 |
| CFSW228 | 1 | Metacaspase-like cysteine peptidase (C14 family, Clan CD) | 1.70E-55 |
| CFSW30 | 3 | Carboxypeptidase B | 4.30E-80 |
| CFSW195 | 2 | C-type lysozyme-1 | 1.30E-26 |
| CFSW457 | 2 | C-type lysozyme-3 | 9E-75 |
| CFSW576 | 1 | C-type lysozyme-2 | 6.30E-37 |
| CFSW1341 | 1 | I-type lysozyme | 1.00E-44 |
| CFSW1263 | 1 | P-type lysozyme | 2.70E-26 |
| CFSW1001 | 1 | Lysosomal Pro-X carboxypeptidase | 2.10E-100 |
| CFSW1405 | 3 | Prolixicin antimicrobial peptide | 1.60E-09 |
| CFSW277 | 6 | Termicin | 3.50E-24 |
| CFSW453 | 3 | Thaumatin-like protein | 2.90E-59 |
| CFSW23 | 4 | Thaumatin-like protein | 3.70E-54 |
| CFSW65 | 1 | Thaumatin-like protein | 8.20E-50 |
