# Supplementary material for: Immune-Related Transcriptome of Coptotermes formosanus Shiraki Workers: The Defense Mechanism
Source: PLoS One. 2013 Jul 16;8(7):e69543. doi: 10.1371/journal.pone.0069543 (PMC3712931; doi:10.1371/journal.pone.0069543)
Supplement: Table S8 — Other immune-related expressed sequences identified from the cDNA library of C. formosanus Shiraki based on sequence similarity ( E ≤10−5). (DOC) [file pone.0069543.s008.doc]

**Table S8. Other immune-related expressed sequences identified from the cDNA library of *C. formosanus* Shiraki based on sequence similarity (*E* ≤ 10^-5^).**

| **Cluster ID** | **No. of Sequences** | **Annotation** | ***E*-value** |
| --- | --- | --- | --- |
| CFSW957 | 1 | Adhesin-like protein (ALP) | 5.20E-11 |
| CFSW517 | 1 | Aspartate aminotransferase (AST) | 1.30E-98 |
| CFSW668 | 1 | ATG4 autophagy related 4 homolog a | 3.5E-16 |
| CFSW311 | 1 | Calponin/Transgelin | 3.3E-88 |
| CFSW497 | 1 | Capping protein (actin filament) muscle Z-line, alpha 2 (CAPZA2) | 1.3E-43 |
| CFSW1264 | 1 | Class B secretin-like G-protein coupled receptor GPRmth5 | 1.50E-32 |
| CFSW118 | 1 | Clathrin coat assembly protein ap17 | 4.50E-77 |
| CFSW1069 | 2 | Clathrin coat assembly protein AP19 | 1.6E-77 |
| CFSW799 | 1 | Cysteine-rich protein 1 (CRP1) | 2.70E-32 |
| CFSW1026 | 1 | Deoxyribonuclease II (DNase II) | 2.0E-45 |
| CFSW764 | 1 | Dipeptidyl peptidase 4 | 1.00E-80 |
| CFSW442 | 1 | DsbA oxidoreductase | 1.40E-36 |
| CFSW590 | 1 | Elongation factor 1-alpha | 1.6E-111 |
| CFSW1025 | 1 | Elongation factor 1-alpha | 9.8E-136 |
| CFSW419 | 3 | Enolase | 1.10E-168 |
| CFSW1136 | 2 | Enolase | 5.7E-108 |
| CFSW482 | 2 | Eukaryotic translation initiation factor 3 | 8.60E-30 |
| CFSW1128 | 2 | Eukaryotic translation initiation factor x-chromosomal | 4.80E-52 |
| CFSW1149 | 1 | Eukaryotic translation initiation factor 3 | 6.40E-127 |
| CFSW1254 | 1 | Eukaryotic translation initiation factor 3 | 1.10E-113 |
| CFSW1276 | 1 | Eukaryotic translation initiation factor 3 | 3.30E-133 |
| CFSW1366 | 1 | Eukaryotic translation initiation factor 4 | 1.70E-34 |
| CFSW1444 | 2 | Eukaryotic translation initiation factor 4e | 8.40E-42 |
| CFSW56 | 2 | Eukaryotic translation initiation factor 4e | 1.90E-37 |
| CFSW435 | 1 | Eukaryotic translation initiation factor 4e | 5.80E-34 |
| CFSW248 | 1 | Exosome component 5 | 3.3E-88 |
| CFSW1426 | 3 | Ferritin | 7.40E-59 |
| CFSW6 | 23 | Ferritin light chain | 4.00E-65 |
| CFSW1502 | 8 | Ferritin heavy chain | 2.3E-69 |
| CFSW1477 | 1 | Ferritin heavy chain | 7.3E-75 |
| CFSW1465 | 1 | Fibrinolytic enzyme | 1.9E-35 |
| CFSW574 | 1 | Fibrinolytic enzyme | 3.9E-44 |
| CFSW80 | 1 | Fimbrin | 8.80E-64 |
| CFSW1171 | 1 | Fimbrin plastin | 2.3E-120 |
| CFSW1098 | 1 | Fk506-binding protein (FKBP) | 1.0E-87 |
| CFSW416 | 3 | Fk506-binding protein (FKBP) | 1.5E-22 |
| CFSW1449 | 1 | GADD45 (CG11086-PA) | 1.00E-20 |
| CFSW691 | 1 | Glycoprotein 2 (GP2) | 9.20E-05 |
| CFSW1239 | 1 | Goliath related E3 Ubiquitin Ligase (GREUL) | 6.2E-79 |
| CFSW172 | 4 | Heat shock protein | 5.1E-68 |
| CFSW433 | 1 | Heat shock protein 90 | 2.8E-10 |
| CFSW1199 | 1 | Heat shock protein 40 | 1.8E-109 |
| CFSW1332 | 1 | Heat shock protein | 2.2E-151 |
| CFSW53 | 1 | Hemocyanin subunit type 1 precursor | 1.0E-40 |
| CFSW1418 | 1 | Hexamerin I | 9.3E-50 |
| CFSW1382 | 1 | Histone H2A.V-like | 1.9E-54 |
| CFSW824 | 1 | Histone H2B | 1.8E-38 |
| CFSW623 | 1 | Hyaluronidase | 1.0E-31 |
| CFSW461 | 1 | Hypervariable *Bacillus* group-specific protein | 1.9E-10 |
| CFSW1310 | 1 | Innexin 2 | 8.7E-104 |
| CFSW546 | 1 | Isoform cra_b | 1.1E-16 |
| CFSW1326 | 1 | Juvenile hormone-inducible | 1.3E-27 |
| CFSW487 | 1 | Leucyl aminopeptidase | 3.00E-07 |
| CFSW372 | 1 | Lumbrokinase-3 | 1.70E-07 |
| CFSW1390 | 1 | Metallophosphoesterase 1 | 1.0E-10 |
| CFSW1022 | 2 | Mitochondrial complement component 1Q subcomponent-binding protein precursor | 2.4E-78 |
| CFSW425 | 1 | MOB1/phocein family protein | 1.00E-24 |
| CFSW1439 | 1 | Mucin-like peritrophin | 8.7E-33 |
| CFSW1052 | 1 | Myelin protein expression factor | 1.6E-42 |
| CFSW135 | 1 | Neuropeptide F (NPF) | 5.60E-33 |
| CFSW55 | 2 | NOP16 nucleolar protein homolog | 4.4E-119 |
| CFSW375 | 1 | Omega gliadin | 1.10E-08 |
| CFSW1115 | 2 | Peritrophin-like protein | 5.0E-31 |
| CFSW511 | 1 | Phosphatidylinositol 3-kinase catalytic subunit type-3 (PIK3C3) | 2.0E-53 |
| CFSW947 | 1 | Phosphoglycerate kinase (PGK) | 2.1E-111 |
| CFSW1336 | 1 | Phosphoglycerate kinase (PGK) | 2.1E-90 |
| CFSW264 | 1 | Proteasome subunit alpha type-2 | 4.40E-119 |
| CFSW528 | 1 | Proteasome (macropain) alpha-6 | 5.80E-116 |
| CFSW402 | 1 | Proteasome subunit beta type-6 | 7.40E-88 |
| CFSW427 | 3 | Proteasome subunit beta-7 | 2.20E-108 |
| CFSW965 | 1 | Protein disulfide isomerase (PDI) | 2.8E-52 |
| CFSW1082 | 3 | Protein disulfide-isomerase A3 (PDIA3) | 3.9E-96 |
| CFSW1380 | 1 | Protein kinase C and casein kinase substrate in neurons **(PACSIN)** | 7.1E-54 |
| CFSW394 | 1 | Protein takeout (JHBP) | 2.3E-54 |
| CFSW739 | 2 | Protein takeout (JHBP) | 4.3E-24 |
| CFSW328 | 1 | Protein takeout (JHBP) | 3.70E-55 |
| CFSW532 | 1 | Protein takeout (JHBP) | 6.1E-45 |
| CFSW445 | 1 | Ricin B Lectin | 1.1E-13 |
| CFSW667 | 1 | Ricin B Lectin | 2.6E-14 |
| CFSW1461 | 2 | Ricin B Lectin | 3.1E-20 |
| CFSW94 | 1 | Saposin-related protein | 3.80E-09 |
| CFSW452 | 3 | SCP-like extracellular domain containing protein 1 | 1E-70 |
| CFSW936 | 1 | SCP-like extracellular domain containing protein 2 | 7E-64 |
| CFSW1186 | 1 | Surface antigen-like | 2.8E-21 |
| CFSW411 | 1 | Syntaxin 13 | 2.40E-62 |
| CFSW310 | 1 | Tetratricopeptide repeat (TPR) protein | 7.30E-10 |
| CFSW1178 | 1 | Teratocyte released chitinase | 2.3E-70 |
| CFSW61 | 1 | Thioredoxin-like protein | 3.90E-48 |
| CFSW437 | 1 | Translation initiation factor subunit | 5.30E-41 |
| CFSW1406 | 4 | Ubiquitin | 2.8E-124 |
| CFSW348 | 6 | Ubiquitin b | 2.8E-106 |
| CFSW1163 | 1 | Ubiquitin carboxyl-terminal hydrolase isozyme l3 | 4.5E-76 |
| CFSW788 | 1 | Ubiquitin conjugating enzyme e2 | 4.5E-43 |
| CFSW115 | 1 | Viral a-type inclusion protein | 4.0E-29 |
| CFSW1033 | 1 | Viral a-type inclusion protein | 4.7E-7 |
| CFSW1042 | 1 | Viral a-type inclusion protein | 1.9E-14 |
| CFSW722 | 1 | Viral a-type inclusion protein | 6.3E-10 |
| CFSW1075 | 4 | Zinc metalloproteinase | 9.2E-62 |
