# Supplementary material for: Immune-Related Transcriptome of Coptotermes formosanus Shiraki Workers: The Defense Mechanism
Source: PLoS One. 2013 Jul 16;8(7):e69543. doi: 10.1371/journal.pone.0069543 (PMC3712931; doi:10.1371/journal.pone.0069543)
Supplement: Table S9 — Identified putative immune-related genes from the Suppression Subtractive Hybridization (SSH) libraries of immunized C. formosanus Shiraki prepared using four microbe types. Footnote of Table S9. - represents absent;+represents present; * indicates that the same gene has also been identified in the normalized cDNA library; ** indicates genes that were quantified using quantitative real time PCR (qRT -PCR). ST: signal transductor; SM: signal modulator; PRR: pattern recognition receptor; O: other immune-related; E: effector. (DOC) [file pone.0069543.s009.doc]

**Table S9. Identified putative immune-related genes from the Suppression Subtractive Hybridization (SSH) libraries of immunized *C. formosanus* Shiraki prepared using four microbe types.**

| **Gene Name** | **Functional categories** | **SHH libraries** | | | |
| --- | --- | --- | --- | --- | --- |
|  |  | ***M. anisopliae*** | ***B. bassiana*** | ***B. thuringiensis*** | ***E. coli*** |
| 14-3-3 protein 1*, ** | ST | + | - | - | - |
| 14-3-3 protein 2* | ST | - | - | + | - |
| α-tubulin-1* | ST | - | - | + | - |
| α-tubulin-2 | ST | + | - | - | - |
| α-tubulin-3 | ST | - | + | + | + |
| α-tubulin-4 | ST | - | - | + | - |
| β-tubulin 5 | ST | + | - | - | - |
| Calmodulin* | ST | + | - | - | - |
| Cofilin tropomyosin-type actin-binding protein* | ST | - | - | + | - |
| EF hand family protein* | ST | + | + | + | - |
| Ejaculatory bulb-specific protein III* | ST | - | - | - | + |
| Four-and-a-half LIM domain protein** | ST | - | + | - | - |
| Phosphoenolpyruvate carboxykinase (PEPCK)* | ST | + | - | - | + |
| Rab GDP-dissociation inhibitor | ST | + | - | - | + |
| Ras family protein* | ST | + | - | - | - |
| Sel1 domain containing protein* | ST | - | - | + | - |
| Serine/threonine protein kinase * | ST | + | - | - | - |
| Small GTP-binding protein* | ST | - | - | + | - |
| Signal peptidase I (Spase I)* | ST | - | - | + | - |
| Chymotrypsin-like protein* | SM | + | - | - | - |
| Serine-rich protein | SM | - | - | + | - |
| Serine protease* | SM | + | + | + | - |
| Apolipophorin-III*, ** | PRR | + | - | - | - |
| β-glucosidase (GH1)* | PRR | - | - | + | - |
| Endo-β-1,4-glucanase (GH9)*, ** | PRR | + | + | + | + |
| Gram negative bacteria binding protein 1** | PRR | - | - | - | + |
| Activating transcription factor | O | - | + | - | - |
| Chemosensory protein | O | + | - | - | + |
| Elongation factor 1 alpha* | O | - | - | + | - |
| Eupolytin | O | - | - | + | - |
| FK506-binding protein (FKBP)* | O | + | - | - | - |
| Ferritin 2** | O | + | + | + | - |
| Ferritin heavy chain* | O | + | - | - | - |
| Ferritin light chain*, ** | O | - | - | - | + |
| Heat shock protein* | O | - | + | + | - |
| Hexamerin I | O | + | - | - | - |
| Macrophage migration inhibitory factor | O | - | - | - | - |
| Peritrophin-like protein | O | + | - | - | - |
| Phosphoglycerate kinase (PGK)* | O | + | - | - | + |
| Proteasome subunit beta type-6* | O | - | - | + | - |
| Protein takeout (JHBP)* | O | + | + | - | + |
| Ricin B lectin* | O | + | - | - | - |
| SCP-like extracellular domain containing protein 2 | O | - | - | - | + |
| SCP-like extracellular domain containing protein 3 | O | - | + | + | - |
| SCP-like extracellular domain containing protein 4 | O | + | - | - | - |
| sol i 3 antigen | O | + | - | - | - |
| Stress-sensitive b | O | - | + | - | + |
| Syntaxin 1a | O | - | + | - | - |
| Teratocyte released chitinase* | O | + | + | + | - |
| Thioredoxin-like protein* | O | - | - | - | + |
| Transferrin** | O | + | - | - | - |
| Viral a-type inclusion protein* | O | - | + | + | + |
| C-type Lysozyme-2*, ** | E | - | + | + | - |
| C-type Lysozyme-4*, ** | E | - | + | - | - |
| Cathepsin D*, ** | E | - | + | - | - |
| Thaumatin-like protein*, ** | E | + | - | - | + |
| Total |  | 27 | 17 | 22 | 15 |

- represents absent; + represents present; * indicates that the same gene has also been identified in the normalized cDNA library; ** indicates genes that were quantified using quantitative real time PCR (qRT -PCR). ST: signal transductor; SM: signal modulator; PRR: pattern recognition receptor; O: other immune-related; E: effector.
